# Omnibus test for normality based on the Edgeworth expansion

**DOI:** 10.1371/journal.pone.0233901

**Published:** 2020-06-11

**Authors:** Agnieszka Wyłomańska, D. Robert Iskander, Krzysztof Burnecki

**Affiliations:** 1 Faculty of Pure and Applied Mathematics, Hugo Steinhaus Center, Wroclaw University of Technology, Wroclaw, Poland; 2 Department of Biomedical Engineering, Faculty of Fundamental Problems of Technology, Wroclaw, Poland; Universita degli Studi di Genova, ITALY

## Abstract

Statistical inference in the form of hypothesis tests and confidence intervals often assumes that the underlying distribution is normal. Similarly, many signal processing techniques rely on the assumption that a stationary time series is normal. As a result, a number of tests have been proposed in the literature for detecting departures from normality. In this article we develop a novel approach to the problem of testing normality by constructing a statistical test based on the Edgeworth expansion, which approximates a probability distribution in terms of its cumulants. By modifying one term of the expansion, we define a test statistic which includes information on the first four moments. We perform a comparison of the proposed test with existing tests for normality by analyzing different platykurtic and leptokurtic distributions including generalized Gaussian, mixed Gaussian, *α*-stable and Student’s *t* distributions. We show for some considered sample sizes that the proposed test is superior in terms of power for the platykurtic distributions whereas for the leptokurtic ones it is close to the best tests like those of D’Agostino-Pearson, Jarque-Bera and Shapiro-Wilk. Finally, we study two real data examples which illustrate the efficacy of the proposed test.

## Introduction

Testing the hypothesis of normality is one of the fundamental procedures of the statistical analysis. There is a large number of normality tests. Some of them such as the *χ*^2^ goodness-of-fit test [1] with its variants, the Kolmogorov-Smirnov (KS) one-sample cumulative probability test [2], the Shapiro-Wilk (SW) test [3], D’Agostino-Pearson (DP) test [4] and Jarque-Bera (JB) test [5] are nowadays considered classical. These tests are based on comparing the distribution of the observed data to the expected distribution (*χ*^2^), on measuring the distance between the empirical and analytical distribution function (KS), on taking into account some transformations of moments of the data like skewness and kurtosis (DP and JB), or on calculating some function of order statistics (SW). Other tests based on the empirical distribution function, less widespread, include the Kuiper test [6], Watson test [7], Cramer-von Mises (CvM) test [8], and the Anderson-Darling (AD) test [9]. From other testing techniques, let us mention ideas based on the empirical characteristic function [10], on the dependence between moments that characterizes normal distributions [11], or on the Noughabi’s entropy estimator [12].

The Edgeworth series can be used to expand an arbitrary probability distribution in terms of its cumulants. So far, the Edgeworth expansion has been utilized to design a score test for normality of errors in a regression model [13], design a normality test for the probit model [14], and to design a normality test against a specific alternative, such as the logistic distribution [15].

There have been also attempts in the literature to provide a one-sample statistical test of normality for data in a broader setting like in a general Hilbert space [16]. Despite this verity, there have been continuing efforts to develop tests for the departure of a random sample from normality that could be considered omnibus, [17, 18, 19, 20, 21, 22], that is, to be able to reject the null hypothesis of normality with high power for a wide range of alternatives.

Many of the normality tests consider evaluating the third and fourth order moments and, hence, the power of such tests depends on whether a symmetric or skewed alternative is being considered. In general, it is expected that symmetric alternatives or those with small amount of skew are more difficult to differentiate from the null hypothesis of normality than those alternatives characterized with a large skew [20]. The fourth order moment, most commonly used in the form of kurtosis, has less obvious effect on the performance of a normality test, but here it is also important whether the distribution is leptokurtic or platykurtic [23, 24], that is, whether its kurtosis is larger or smaller than that of the normal distribution, respectively.

There are many signal processing applications in which the underlying distribution of the data is leptokurtic [25, 26], while those phenomena that can be modeled with a platykurtic distribution, with the exception of the uniform distribution, are less present [27]. Consequently, normality tests dedicated to platykurtic alternatives are scarce. Nevertheless, some effort has been made to improve the performance of a normality tests across the range of symmetric platykurtic alternatives [28].

The aim of this work is to develop a novel test for normality of omnibus character that could outperform the classical tests for the case of platykurtic symmetric alternatives.

The paper is structured as follows. First, we derive a test statistic based on the second term of the Edgeworth expansion which incorporates information on both the skewness and kurtosis. In the next part we establish the main results. We formally construct a statistical test on normality and provide information on critical values of the test. Next, we analyze the power of the test by Monte Carlo simulations. We take into account four symmetric distributions which can be very close to the normal law, namely the generalized Gaussian, mix Gaussian distributions, *α*-stable and Student’s *t*-distributions. The former two serve as examples of platykurtic distributions, whereas the latter two are classical leptokurtic probability laws. We compare the results with the power of the classical normality tests. Finally, we study two real data examples taken from a collection of over 1300 datasets that were originally distributed alongside the statistical software environment R and some of its add-on packages. The examples illustrate the efficacy of the proposed test. The findings of the paper are summarized in the last section.

## Derivation of the new test statistic based on Edgeworth expansion

Let *X*_1_, *X*_2_, …, *X*_*N*_ be a random sample from the distribution with finite mean *μ* = *E*(*X*_1_). We define the arithmetic mean ${\bar{X}}_{n}=\frac{1}{n}\sum_{i=1}^{n}X_{i}$, *n* = 1, 2, …, *N* and the standardized mean by

$$\begin{array}{c} T_{n}=\sqrt{n}({\bar{X}}_{n}-\mu ){\left(\frac{1}{n}\sum_{i=1}^{n}X_{i}^{2}-{\bar{X}}_{n}^{2}\right)}^{-1/2}. \end{array}$$ (1)

The Edgeworth expansion is a series that approximates a probability distribution in terms of its cumulants [29]. For random variables *X*_*i*_, *i* = 1, 2, …, *N*, with finite *k*th moment it has the following form

$$\begin{array}{c} F_{n}\left(y\right)=P\left(T_{n}\leq y\right)=\Phi \left(y\right)+\sum_{i=1}^{k}H_{i}\left(y\right)+o\left(n^{-k/2}\right), \end{array}$$ (2)

where

$$\begin{array}{c} H_{i}\left(y\right)=n^{-i/2}P_{i}\left(y\right)\phi \left(y\right). \end{array}$$ (3)

In Eqs (2) and (3), Φ(*y*) and *ϕ*(*y*) stand for the cumulative distribution function (CDF) and the probability density function (PDF) of a standard normal distribution, respectively, and *P*_*i*_(*y*) is an appropriate Hermite polynomial of degree 3*i* − 1 [29]. The coefficients in *P*_*i*_(⋅) are expressed in terms of appropriate moments of the random variable *X*_1_. For instance, the first two polynomials have the following form

$$\begin{array}{ccc} P_{1}\left(y\right) & = & \frac{1}{6}\tau \left(2y^{2}+1\right), \end{array}$$ (4)

$$\begin{array}{ccc} P_{2}\left(y\right) & = & -y(\frac{1}{18}\tau^{2}\left(y^{4}+2y^{2}-3\right)-\frac{1}{12}\kappa \left(y^{2}-3\right)+\frac{1}{4}\left(y^{2}+3\right)), \end{array}$$ (5)

where *τ* = *E*(*X*_1_ − *μ*)^3^(*VarX*_1_)^−3/2^ and *κ* = *E*(*X*_1_−*μ*)^4^(*VarX*_1_)^−2^−3 are the skewness and excess kurtosis (in this paper in the figures also called, in short, kurtosis) of the random variable *X*_1_.

Let us now concentrate on the statistic *T*_*n*_ for *n* = 2 (see Eq (1)), which depends only on two random variables *X*_1_ and *X*_2_. Such a choice of *n* allows to have many realizations of the statistic for one sufficiently long trajectory by dividing the data into blocks of length 2. The choice of *n* = 2 seems to be the most optimal, however in order to prove this, in the simulation study we present the comparison between the results obtained in case of *n* = 2 and *n* = 3. By Eq (2) the CDF of the *T*_2_ statistic can be approximated by

$$\begin{array}{c} F_{2}\left(y\right)=P\left(T_{2}\leq y\right)≅\Phi \left(y\right)+\sum_{i=1}^{k}H_{i}\left(y\right). \end{array}$$ (6)

Also, (*n* + 1)/*nT*_2_ has a Student’s *t*-distribution with 1 degree of freedom when *X*_1_ follows the normal distribution. For practical reasons we assume *k* = 2. The functions *H*_*i*_(*y*), *i* = 1, 2 contain the information about the deviation of the *T*_2_ distribution from the standard normal law. If the distribution underlying the random sample is close to normal we expect the deviations (hence the functions) to be smaller than those for non-normal distributions. They can be also called corrections to the normal distribution since the CDF of *T*_2_ is approximated by the CDF of the standard normal distribution corrected by those functions.

Let us now define two statistics $H_{1}^{max}$ and $H_{2}^{max}$ as the maxima of the functions *H*_1_(*y*) and *H*_2_(*y*), respectively, calculated over the *y* values at which the empirical CDF of *T*_2_ changes, hence the *T*_2_ values. This is similar to calculating the Kolmogorov-Smirnov statistic. Let

$$\begin{array}{c} {\bar{X}}_{i,i+1}=\frac{1}{2}\sum_{j=i}^{i+1}X_{j} \end{array}$$ (7)

and

$$\begin{array}{c} T_{2}^{i}=\sqrt{2}\left({\bar{X}}_{i,i+1}-\mu \right){\left(\frac{1}{2}\sum_{j=i}^{i+1}X_{j}^{2}-{\left({\bar{X}}_{i,i+1}\right)}^{2}\right)}^{-1/2},\;i=1,2,\ldots ,N-1. \end{array}$$ (8)

This approach is arbitrary and other ways of arranging the random sample into blocks of length two are permitted. Then

$$\begin{array}{c} H_{i}^{max}=\underset{y}{\text{max}}|H_{i}\left(y\right)|=\frac{1}{2}\underset{y}{\text{max}}|P_{i}\left(y\right)\phi \left(y\right)|,\;\;\;\;i=1,2, \end{array}$$ (9)

where $y=\{T_{2}^{2i+1}:\;i=0,1,\ldots ,\left\lfloor N/2\right\rfloor -1\}$.

**Fig 1:**
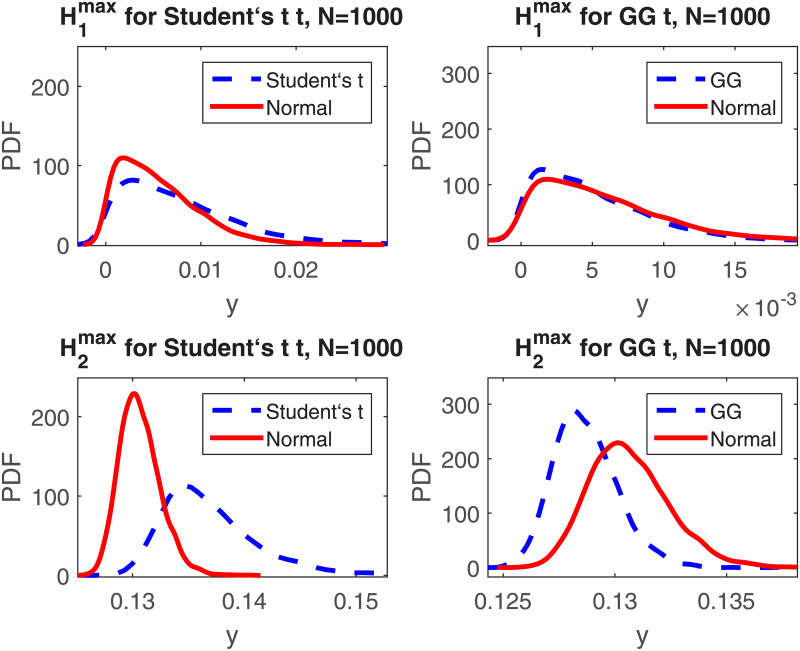
Empirical PDFs of $H_{1}^{max}$ and $H_{2}^{max}$ for the Student’s *t*-distribution with *ν* = 16 degrees of freedom (left panels) and generalized Gaussian (GG) distribution with *μ* = 1, *β* = 0.2, *ρ* = 2.2 (right panels) with corresponding empirical PDFs obtained for the standard normal distribution.

To ascertain whether the test statistic $H_{i}^{max}$, *i* = 1, 2, is sensitive to deviations from normality we perform Monte Carlo simulations for two non-normal distributions described in more detail in the Appendix. They are the generalized Gaussian (GG) distribution with parameters *μ* = 1, *β* = 0.2, and *ρ* = 2.2 corresponding to a case of platykurtic distribution and the Student’s *t*-distribution with *ν* = 16 degrees of freedom, corresponding to the case of a leptokurtic distribution. For *M* simulated samples *x*_1_, *x*_2_, ⋯, *x*_*N*_ of size *N* we calculate ⌊*N*/2⌋ values of the *T*_2_ statistic and evaluate the maximum of *H*_*i*_(*y*), *i* = 1, 2, over all values of the standardized means *T*_2_ obtained for a given sample according to Eq (9). As a result, we obtain *M* realizations of $H_{i}^{max}$, *i* = 1, 2. In Fig 1 we present a comparison of the empirical PDFs of $H_{i}^{max}$, *i* = 1, 2, for the two considered distributions and the corresponding empirical PDFs obtained for the standard normal distribution. The empirical PDFs were constructed as kernel density estimators [30]. In the simulations we considered *N* = 1000 and *M* = 5000.

We can observe that $H_{1}^{max}$ is less sensitive to deviations from normality than the $H_{2}^{max}$ statistic. This effect is visible for both analyzed non-normal distributions. Therefore, we propose a test for normality based on the $H_{2}^{max}$ *test statistic*

$$\begin{array}{c} H_{2}^{max}=\underset{y}{\text{max}}|H_{2}\left(y\right)|=\frac{1}{2}\underset{y}{\text{max}}|P_{2}\left(y\right)\phi \left(y\right)|, \end{array}$$ (10)

where $y=\{T_{2}^{2i+1}:\;i=0,1,\ldots ,N/2-1\}$.

## Testing for normality

### Construction of the test

In our statistical test the null hypothesis (*H*_0_) is that the data come from a normal distribution with an unknown mean and variance. The alternative hypothesis (*H*_1_) is that the data set does not come from such a distribution. The test statistic is given by formula (10).

As explained in the previous section, for sample data *x*_1_, *x*_2_, ⋯, *x*_*N*_, first, we calculate averages ${\bar{x}}_{i,i+1}=\frac{1}{2}\sum_{j=i}^{i+1}x_{j}$. Then, we calculate values of the $T_{2}^{2i+1}$ statistic defined by Eq (8) by taking every two consecutive observations (without overlapping). In consequence, from the sample of size *N* we obtain ⌊*N*/2⌋ values of the $T_{2}^{2i+1}$ statistic. Finally, we calculate $H_{2}^{max}$ according to Eq (10) by taking the maximum over the $T_{2}^{2i+1}$ statistic values. It is worth to mention that in the formulas for *T*_2_ and $H_{2}^{max}$ we replace *μ*, *τ* and *κ* coefficients by the sample mean, skewness and excess kurtosis, respectively, calculated for the whole series *x*_1_, *x*_2_, ⋯, *x*_*N*_.

**Fig 2:**
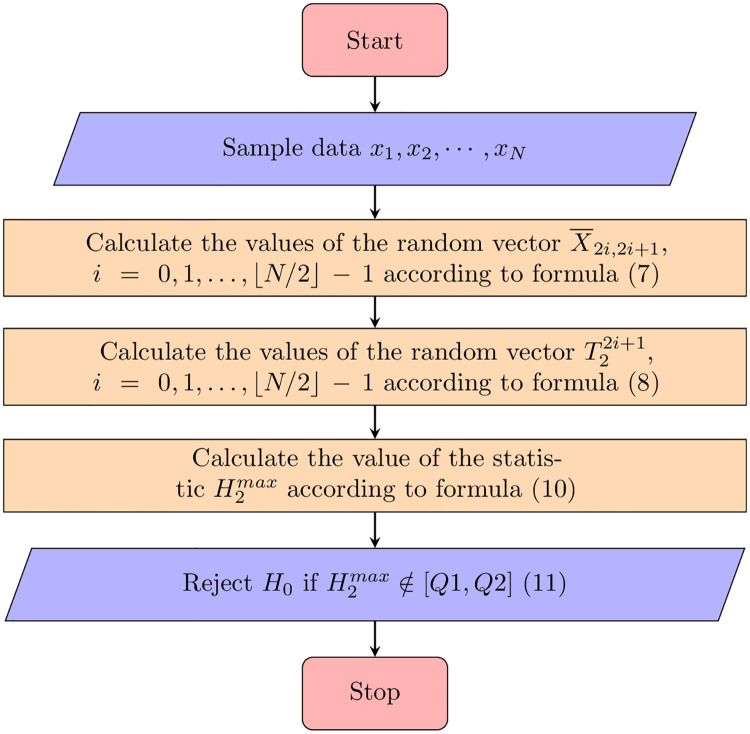
Schema 1. Schematic algorithm of the testing procedure.

We reject the *H*_0_ hypothesis if the test statistic is extreme, either larger than an upper critical value or smaller than a lower critical value at a given significance level *c*. The procedure of testing is summarized in Fig 2 Schema 1.

In order to construct the critical region we advocate the use of Monte Carlo simulations. We simulate *M* trajectories of size *N* of independent identically distributed (i.i.d.) random variables from the standard normal distribution. As a result we obtain a matrix of size *M* × *N*. For each trajectory we calculate the $H_{2}^{max}$ statistic. The critical region is defined as

$$\begin{array}{c} {}[Q1,Q2], \end{array}$$ (11)

where *Q*1 and *Q*2 are are empirical quantiles of order *c*/2 and 1 − *c*/2, respectively, calculated from the *M* values of $H_{2}^{max}$. The *Q*1 and *Q*2 are called lower and upper critical values.

The critical values for five different sample data sizes (*N* = 20, 50, 100, 200, 1000) based on *M* = 5000 Monte Carlo simulations for two selected significance levels *c* = 5% and *c* = 1% are presented in Table 1 in the Appendix.

**Table 1:** The lower and upper critical values *Q*1 and *Q*2 for sample sizes 20, *N* = 50, 100, 200 and 1000 and two exemplary significance levels *c*: 0.05 and 0.01. The critical values are calculated based on the 5000 Monte Carlo simulations of standard normal distributed samples.

	*Q*1, *c* = 0.05	*Q*2, *c* = 0.05	*Q*1, *c* = 0.01	*Q*2, *c* = 0.01
*N* = 20	0.0848	0.1694	0.0636	0.2060
*N* = 50	0.1172	0.1546	0.1099	0.1747
*N* = 100	0.1229	0.1466	0.1210	0.1604
*N* = 200	0.1249	0.1417	0.1238	0.1486
*N* = 1000	0.1276	0.1346	0.1269	0.1364

### Power simulation study

The power of the test is the probability to reject the null hypothesis *H*_0_ when the alternative *H*_1_ is true. The power is an important characteristic of any statistical test. In our case the power of the test is defined as follows:

$$\begin{array}{c} power=P_{H_{1}}[H_{2}^{max}\notin \left[Q1,Q2\right]], \end{array}$$ (12)

where *Q*1 and *Q*2 are lower and upper critical values, respectively. In our study, for all considered cases we calculate the power of the test by using Monte Carlo simulations. More precisely, for a given sample size *N* we simulate *M* independent trajectories from considered distribution. For each trajectory the value of $H_{2}^{max}$ test statistics is calculated and we check if the value falls into the critical region constructed for a given significance level *c*. The power of the test is evaluated as the fraction of trajectories for which the value of $H_{2}^{max}$ is larger than an upper critical value *Q*2 or smaller than a lower critical value *Q*1.

**Fig 3:**
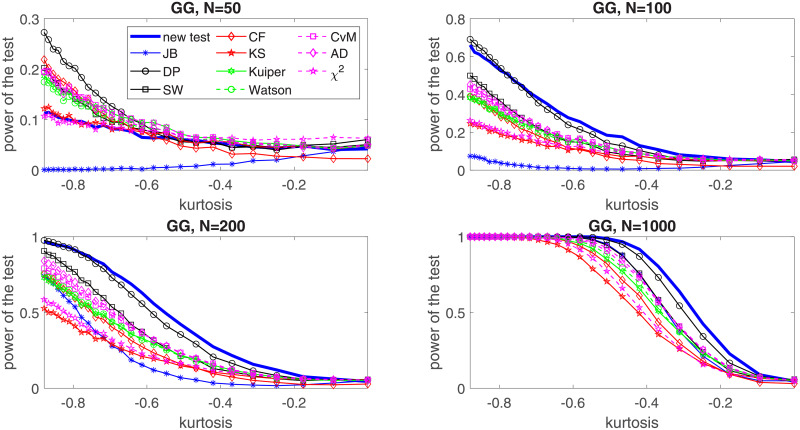
Power of the introduced test and standard tests for normality for different sample sizes: *N* = 50, *N* = 100; *N* = 200 and *N* = 1000 for the generalized Gaussian distribution with respect to the excess kurtosis. Powers were calculated on the basis of 5000 simulations. The significance level is equal to 5%.

**Fig 4:**
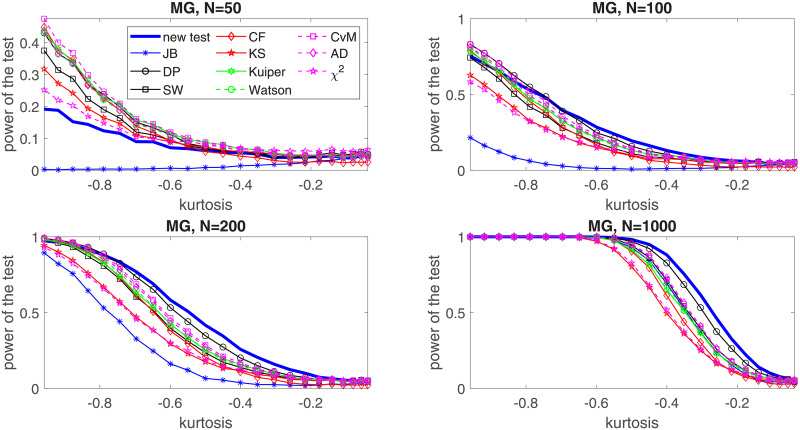
Power of the introduced test and standard tests for normality for different sample sizes: *N* = 50, *N* = 100; *N* = 200 and *N* = 1000, for the mixed Gaussian distribution with respect to the excess kurtosis. Powers were calculated on the basis of 5000 simulations. The significance level is equal to 5%.

**Fig 5:**
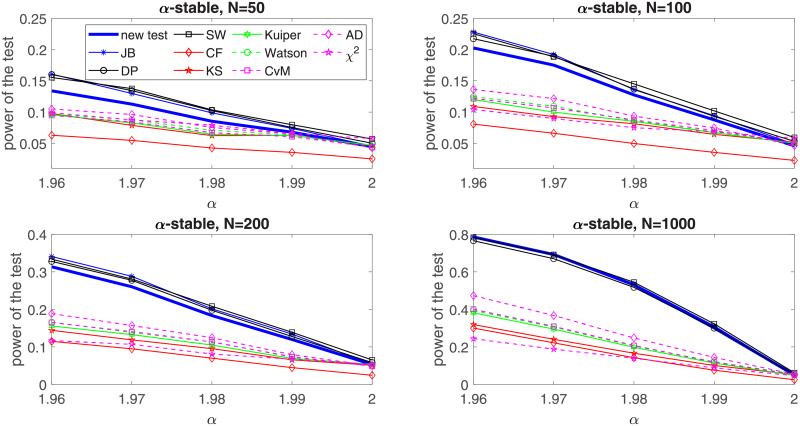
Power of the introduced test and standard tests for normality for different sample sizes: *N* = 50, *N* = 100; *N* = 200 and *N* = 1000, for the *α*-stable distribution with respect to the parameter. Powers were calculated on the basis of 5000 simulations. The significance level is equal to 5%.

**Fig 6:**
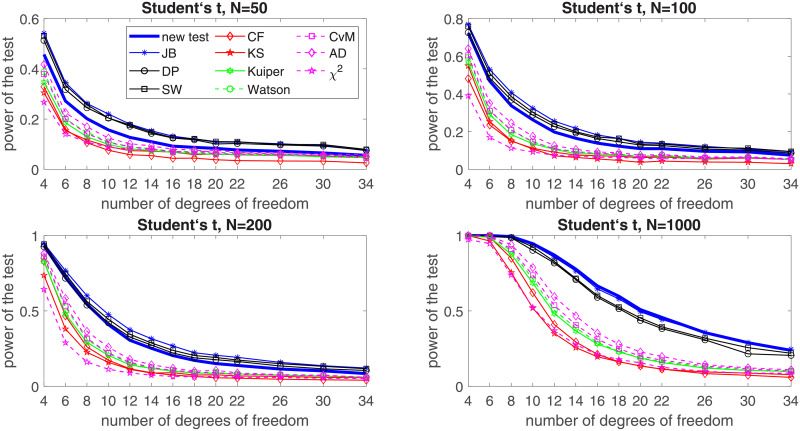
Power of the introduced test and standard tests for normality for different sample sizes: *N* = 50, *N* = 100; *N* = 200 and *N* = 1000, for the Student’s t-distribution with respect to the number of degrees of freedom. Powers were calculated on the basis of 5000 simulations. The significance level is equal to 5%.

In the following, we perform a simulation study for four selected distributions: two of them belong to the platykurtic class of distributions and two—to the leptokurtic. In the first group we choose the generalized Gaussian and the mixed Gaussian distributions, for which the JB test was found to perform poorly [24]. In the second group, *α*-stable and Student’s *t* distributions are considered. In order to show the effectiveness of the proposed test for each considered distribution we examine five values of the sample size, namely *N* = 20, *N* = 50, *N* = 100, *N* = 200 and *N* = 1000. We assume the significance level *c* = 0.05. For the implementation of the test and the simulation study we used MATLAB R2019a. Simulations were performed on Intel(R) Core(TM) i7-7500U CPU @ 2.7 GHz. In this section we graphically illustrate the results for *N* = 50, 100, 200, 1000. The powers for all considered sizes and the graphical comparison of the *N* = 20 and *N* = 50 cases are presented in the Appendix, see Tables 2–21 and Figs 3–6.

**Fig 7:**
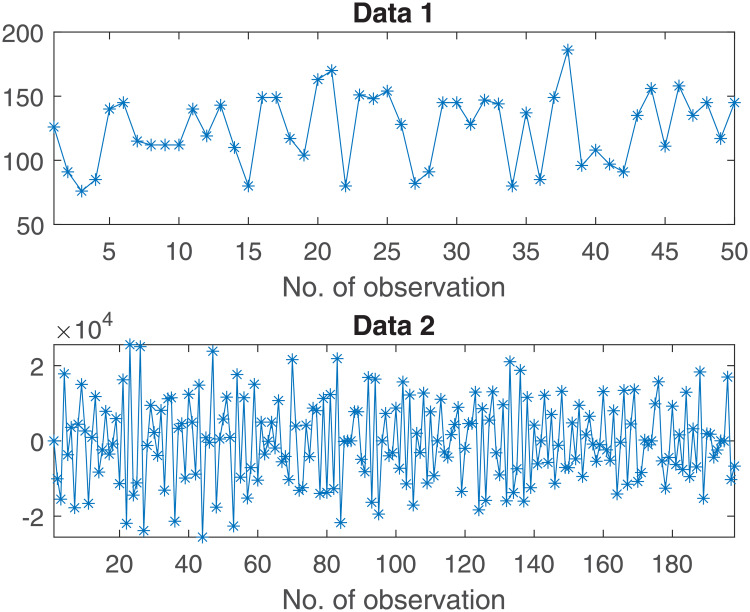
The oil investment (Data1) and differentiated real earnings (Data2) datasets.

In Fig 7 we study the power of the proposed test for normality for the generalized Gaussian distribution. For the distribution we assume that *μ* = 1, *β* = 0.2 and analyze the power by changing the *ρ* parameter. As it is described in the Appendix, in this case the *ρ* parameter controls if the generalized Gaussian distribution belongs to the leptokurtic or platykurtic class of distributions. Since there is one to one correspondence between the *ρ* parameter and the excess kurtosis (see formula (14)), we present the power of the test with respect to the excess kurtosis. It is compared with the power of the most common tests for normality, namely JB, DP, SW, Kuiper, Watson, CvM, AD, *χ*^2^ and the test of Zoubir and Arnold [22], based on the empirical characteristic function (CF), that was shown to perform well for smaller sample sizes. We can see that the proposed test is clearly superior to other tests for *N* ≥ 200 and that for *N* = 100 it shares this superiority to other tests with the DP test, which, on the other hand, performs best among considered tests for sample size *N* = 50 and very small kurtosis values. We can also observe that the least performing tests in this study are the KS test, the *χ*^2^ test, and (surprisingly) the JB test, especially for short samples.

**Table 2:** Comparison of the powers of the tests for normality for GG distribution and *N* = 20. The following tests are taken under consideration: the new test proposed in this paper, Jarque-Bera (JB) test [5], D’Agostino-Pearson (DP) test [4], Shapiro-Wilk (SW) test [3], test based on the empirical characteristic function (CF) [22], Kolmogorov-Smirnov (KS) test [2], Kuiper test [6], Watson test [7], Cramer-von Mises (CvM) test [8], Anderson-Darling (AD) test [9] and *χ*^2^ goodness-of-fit test [1].

*kurtosis*	*new test*	*JB*	*DP*	*SW*	*CF*	*KS*	*Kuiper*	*Watson*	*CvM*	*AD*	*χ*^2^
0.0000	0.0446	0.0474	0.0540	**0.0594**	0.0492	0.0514	0.0488	0.0520	0.0524	0.0484	0.0000
-0.0934	0.0458	0.0414	0.0450	**0.0546**	0.0448	0.0476	0.0472	0.0478	0.0446	0.0448	0.0000
-0.1753	0.0468	0.0386	0.0458	0.0506	**0.0526**	0.0502	0.0468	0.0424	0.0436	0.0412	0.0000
-0.2475	0.0454	0.0304	0.0376	0.0470	**0.0552**	0.0484	0.0498	0.0422	0.0426	0.0424	0.0000
-0.3116	0.0438	0.0308	0.0408	0.0498	**0.0596**	0.0478	0.0506	0.0508	0.0494	0.0474	0.0000
-0.3688	0.0434	0.0262	0.0360	0.0480	**0.0594**	0.0476	0.0470	0.0446	0.0440	0.0438	0.0000
-0.4202	0.0472	0.0228	0.0356	0.0466	**0.0656**	0.0430	0.0490	0.0454	0.0406	0.0416	0.0000
-0.4666	0.0484	0.0198	0.0300	0.0484	**0.0676**	0.0484	0.0518	0.0530	0.0504	0.0480	0.0000
-0.5086	0.0446	0.0206	0.0326	0.0490	**0.0720**	0.0488	0.0576	0.0584	0.0542	0.0544	0.0000
-0.5468	0.0468	0.0156	0.0314	0.0482	**0.0724**	0.0516	0.0568	0.0568	0.0526	0.0494	0.0000
-0.5816	0.0514	0.0126	0.0252	0.0460	**0.0776**	0.0490	0.0560	0.0554	0.0498	0.0464	0.0000
-0.6135	0.0454	0.0088	0.0266	0.0472	**0.0840**	0.0488	0.0582	0.0548	0.0516	0.0508	0.0000
-0.6428	0.0468	0.0116	0.0280	0.0514	**0.0912**	0.0562	0.0620	0.0558	0.0546	0.0540	0.0000
-0.6698	0.0468	0.0098	0.0322	0.0526	**0.0976**	0.0524	0.0636	0.0588	0.0554	0.0566	0.0000
-0.6948	0.0482	0.0096	0.0286	0.0504	**0.1038**	0.0500	0.0602	0.0618	0.0556	0.0536	0.0000
-0.7179	0.0490	0.0104	0.0294	0.0566	**0.1002**	0.0538	0.0632	0.0628	0.0568	0.0568	0.0000
-0.7394	0.0522	0.0074	0.0298	0.0530	**0.1086**	0.0544	0.0696	0.0610	0.0548	0.0574	0.0000
-0.7593	0.0528	0.0074	0.0336	0.0586	**0.1172**	0.0560	0.0670	0.0714	0.0644	0.0640	0.0000
-0.7779	0.0498	0.0050	0.0276	0.0532	**0.1130**	0.0546	0.0628	0.0636	0.0566	0.0552	0.0000
-0.7953	0.0592	0.0068	0.0344	0.0604	**0.1282**	0.0594	0.0712	0.0728	0.0634	0.0640	0.0000
-0.8116	0.0562	0.0054	0.0330	0.0606	**0.1270**	0.0646	0.0786	0.0752	0.0658	0.0670	0.0000
-0.8268	0.0542	0.0066	0.0378	0.0632	**0.1364**	0.0558	0.0708	0.0710	0.0630	0.0604	0.0000
-0.8411	0.0544	0.0050	0.0332	0.0578	**0.1350**	0.0584	0.0720	0.0698	0.0620	0.0618	0.0000
-0.8546	0.0564	0.0056	0.0368	0.0646	**0.1434**	0.0604	0.0754	0.0752	0.0654	0.0688	0.0000
-0.8672	0.0560	0.0060	0.0388	0.0678	**0.1482**	0.0620	0.0770	0.0746	0.0648	0.0682	0.0000
-0.8792	0.0612	0.0062	0.0398	0.0704	**0.1496**	0.0650	0.0846	0.0804	0.0734	0.0726	0.0000

**Table 3:** Comparison of the powers of the tests for normality for GG distribution and *N* = 50. The following tests are taken under consideration: the new test proposed in this paper, Jarque-Bera (JB) test [5], D’Agostino-Pearson (DP) test [4], Shapiro-Wilk (SW) test [3], test based on the empirical characteristic function (CF) [22], Kolmogorov-Smirnov (KS) test [2], Kuiper test [6], Watson test [7], Cramer-von Mises (CvM) test [8], Anderson-Darling (AD) test [9] and *χ*^2^ goodness-of-fit test [1].

*kurtosis*	*new test*	*JB*	*DP*	*SW*	*CF*	*KS*	*Kuiper*	*Watson*	*CvM*	*AD*	*χ*^2^
0.0000	0.0420	0.0470	0.0506	0.0606	0.0226	0.0484	0.0486	0.0482	0.0482	0.0506	**0.0632**
-0.0934	0.0406	0.0366	0.0452	0.0520	0.0228	0.0418	0.0424	0.0456	0.0444	0.0430	**0.0648**
-0.1753	0.0476	0.0284	0.0472	0.0492	0.0260	0.0488	0.0478	0.0474	0.0476	0.0444	**0.0612**
-0.2475	0.0438	0.0202	0.0400	0.0436	0.0276	0.0452	0.0526	0.0498	0.0518	0.0462	**0.0612**
-0.3116	0.0460	0.0186	0.0460	0.0496	0.0328	0.0466	0.0538	0.0512	0.0536	0.0534	**0.0592**
-0.3688	0.0514	0.0120	0.0448	0.0446	0.0316	0.0548	0.0584	0.0524	0.0554	0.0518	**0.0638**
-0.4202	0.0534	0.0116	0.0564	0.0552	0.0456	0.0584	0.0642	0.0620	0.0654	0.0602	**0.0646**
-0.4666	0.0592	0.0078	0.0552	0.0540	0.0434	0.0528	**0.0648**	0.0598	0.0650	0.0586	0.0644
-0.5086	0.0604	0.0068	0.0612	0.0566	0.0480	0.0590	0.0716	0.0652	**0.0722**	0.0664	0.0706
-0.5468	0.0654	0.0054	0.0726	0.0652	0.0592	0.0742	**0.0852**	0.0788	0.0848	0.0766	0.0750
-0.5816	0.0636	0.0048	0.0768	0.0742	0.0646	0.0720	**0.0942**	0.0822	0.0942	0.0844	0.0774
-0.6135	0.0648	0.0020	0.0922	0.0808	0.0778	0.0754	0.0944	0.0834	**0.0954**	0.0860	0.0782
-0.6428	0.0768	0.0034	0.1006	0.0840	0.0836	0.0794	0.1020	0.0906	**0.1026**	0.0938	0.0778
-0.6698	0.0810	0.0036	**0.1154**	0.0946	0.0964	0.0824	0.1052	0.0980	0.1122	0.1020	0.0836
-0.6948	0.0816	0.0024	**0.1262**	0.0952	0.0964	0.0804	0.1132	0.0992	0.1146	0.1074	0.0846
-0.7179	0.0888	0.0022	**0.1386**	0.1030	0.1122	0.0906	0.1250	0.1096	0.1234	0.1180	0.0934
-0.7394	0.0846	0.0024	**0.1528**	0.1104	0.1190	0.0946	0.1266	0.1130	0.1292	0.1208	0.0806
-0.7593	0.0912	0.0014	**0.1644**	0.1262	0.1408	0.0920	0.1338	0.1264	0.1436	0.1368	0.0886
-0.7779	0.0966	0.0010	**0.1836**	0.1320	0.1410	0.0900	0.1380	0.1272	0.1448	0.1412	0.0930
-0.7953	0.0968	0.0018	**0.2018**	0.1406	0.1570	0.0970	0.1410	0.1330	0.1518	0.1450	0.0952
-0.8116	0.0994	0.0008	**0.2066**	0.1556	0.1666	0.1086	0.1536	0.1456	0.1654	0.1606	0.0940
-0.8268	0.1024	0.0010	**0.2152**	0.1558	0.1748	0.1022	0.1538	0.1374	0.1580	0.1514	0.0964
-0.8411	0.1046	0.0012	**0.2372**	0.1728	0.1806	0.1098	0.1594	0.1548	0.1718	0.1684	0.0932
-0.8546	0.1100	0.0012	**0.2398**	0.1780	0.1904	0.1134	0.1694	0.1606	0.1810	0.1790	0.1032
-0.8672	0.1146	0.0010	**0.2584**	0.1936	0.2020	0.1248	0.1778	0.1728	0.1964	0.1934	0.1088
-0.8792	0.1126	0.0008	**0.2728**	0.2024	0.2190	0.1220	0.1862	0.1746	0.1948	0.1950	0.1070

**Table 4:** Comparison of the powers of the tests for normality for GG distribution and *N* = 100. The following tests are taken under consideration: the new test proposed in this paper, Jarque-Bera (JB) test [5], D’Agostino-Pearson (DP) test [4], Shapiro-Wilk (SW) test [3], test based on the empirical characteristic function (CF) [22], Kolmogorov-Smirnov (KS) test [2], Kuiper test [6], Watson test [7], Cramer-von Mises (CvM) test [8], Anderson-Darling (AD) test [9] and *χ*^2^ goodness-of-fit test [1].

*kurtosis*	*new test*	*JB*	*DP*	*SW*	*CF*	*KS*	*Kuiper*	*Watson*	*CvM*	*AD*	*χ*^2^
0.0000	0.0452	0.0452	0.0492	0.0560	0.0204	0.0484	0.0486	0.0496	0.0476	0.0502	**0.0560**
-0.0934	0.0544	0.0340	0.0488	0.0548	0.0206	0.0514	**0.0548**	0.0534	0.0546	0.0546	0.0546
-0.1753	0.0588	0.0248	0.0512	0.0508	0.0208	0.0510	0.0514	0.0488	0.0510	0.0484	**0.0592**
-0.2475	**0.0712**	0.0158	0.0526	0.0470	0.0256	0.0496	0.0592	0.0532	0.0544	0.0496	0.0650
-0.3116	**0.0810**	0.0104	0.0588	0.0518	0.0304	0.0594	0.0696	0.0624	0.0654	0.0608	0.0648
-0.3688	**0.1080**	0.0090	0.0812	0.0604	0.0346	0.0614	0.0710	0.0670	0.0752	0.0696	0.0726
-0.4202	**0.1298**	0.0074	0.1036	0.0740	0.0520	0.0736	0.0892	0.0868	0.0954	0.0872	0.0806
-0.4666	**0.1758**	0.0052	0.1282	0.0838	0.0572	0.0718	0.1006	0.0892	0.1004	0.0950	0.0920
-0.5086	**0.1844**	0.0060	0.1430	0.0964	0.0700	0.0842	0.1232	0.1128	0.1278	0.1184	0.0970
-0.5468	**0.2188**	0.0060	0.1878	0.1300	0.0922	0.0976	0.1320	0.1310	0.1478	0.1424	0.1050
-0.5816	**0.2560**	0.0070	0.2150	0.1468	0.1088	0.1144	0.1640	0.1478	0.1666	0.1608	0.1280
-0.6135	**0.2772**	0.0094	0.2392	0.1506	0.1164	0.1048	0.1556	0.1498	0.1694	0.1640	0.1210
-0.6428	**0.3118**	0.0124	0.2838	0.1720	0.1448	0.1256	0.1808	0.1662	0.1904	0.1822	0.1336
-0.6698	**0.3496**	0.0118	0.3196	0.2002	0.1610	0.1248	0.1926	0.1844	0.2088	0.2110	0.1456
-0.6948	**0.3804**	0.0138	0.3548	0.2228	0.1814	0.1394	0.2174	0.2016	0.2314	0.2314	0.1502
-0.7179	**0.4096**	0.0196	0.4004	0.2500	0.2022	0.1546	0.2372	0.2314	0.2566	0.2560	0.1762
-0.7394	**0.4474**	0.0240	0.4378	0.2770	0.2164	0.1586	0.2478	0.2356	0.2660	0.2688	0.1808
-0.7593	0.4714	0.0280	**0.4726**	0.3048	0.2410	0.1736	0.2674	0.2642	0.2970	0.2960	0.1776
-0.7779	**0.5074**	0.0322	0.5024	0.3304	0.2670	0.1802	0.2850	0.2744	0.3054	0.3130	0.1958
-0.7953	0.5238	0.0370	**0.5364**	0.3616	0.2804	0.1870	0.2980	0.2894	0.3254	0.3392	0.2054
-0.8116	0.5494	0.0454	**0.5688**	0.3844	0.3080	0.2076	0.3270	0.3180	0.3564	0.3630	0.2162
-0.8268	0.5748	0.0440	**0.5956**	0.4044	0.3280	0.2052	0.3214	0.3222	0.3632	0.3770	0.2254
-0.8411	0.5922	0.0598	**0.6298**	0.4398	0.3432	0.2306	0.3556	0.3472	0.3846	0.4014	0.2292
-0.8546	0.6122	0.0664	**0.6464**	0.4514	0.3648	0.2308	0.3658	0.3658	0.4020	0.4196	0.2470
-0.8672	0.6228	0.0720	**0.6706**	0.4820	0.3794	0.2408	0.3700	0.3752	0.4112	0.4330	0.2476
-0.8792	0.6606	0.0738	**0.6908**	0.4992	0.3878	0.2460	0.3810	0.3896	0.4296	0.4520	0.2632

**Table 5:** Comparison of the powers of the tests for normality for GG distribution and *N* = 200. The following tests are taken under consideration: the new test proposed in this paper, Jarque-Bera (JB) test [5], D’Agostino-Pearson (DP) test [4], Shapiro-Wilk (SW) test [3], test based on the empirical characteristic function (CF) [22], Kolmogorov-Smirnov (KS) test [2], Kuiper test [6], Watson test [7], Cramer-von Mises (CvM) test [8], Anderson-Darling (AD) test [9] and *χ*^2^ goodness-of-fit test [1].

*kurtosis*	*new test*	*JB*	*DP*	*SW*	*CF*	*KS*	*Kuiper*	*Watson*	*CvM*	*AD*	*χ*^2^
0.0000	0.0442	0.0490	0.0526	0.0568	0.0272	0.0548	0.0518	0.0526	0.0534	0.0516	**0.0556**
-0.0934	**0.0604**	0.0340	0.0538	0.0554	0.0222	0.0516	0.0504	0.0530	0.0532	0.0558	0.0564
-0.1753	**0.0754**	0.0222	0.0600	0.0500	0.0242	0.0580	0.0574	0.0558	0.0612	0.0570	0.0652
-0.2475	**0.1204**	0.0162	0.0836	0.0614	0.0378	0.0626	0.0742	0.0652	0.0740	0.0708	0.0724
-0.3116	**0.1576**	0.0204	0.1144	0.0808	0.0514	0.0754	0.0936	0.0910	0.1000	0.0938	0.0862
-0.3688	**0.2156**	0.0258	0.1640	0.1092	0.0730	0.0910	0.1198	0.1098	0.1216	0.1172	0.1014
-0.4202	**0.2738**	0.0340	0.2062	0.1292	0.0964	0.1054	0.1514	0.1446	0.1630	0.1546	0.1158
-0.4666	**0.3514**	0.0542	0.2912	0.1904	0.1334	0.1314	0.1886	0.1766	0.1970	0.1950	0.1446
-0.5086	**0.4218**	0.0690	0.3540	0.2124	0.1588	0.1478	0.2172	0.2118	0.2350	0.2296	0.1646
-0.5468	**0.491**	0.0968	0.4110	0.2646	0.1984	0.1754	0.2586	0.2522	0.2782	0.2788	0.1792
-0.5816	**0.5584**	0.1194	0.4856	0.3136	0.2334	0.1934	0.2958	0.2886	0.3182	0.3164	0.2068
-0.6135	**0.6296**	0.1536	0.5576	0.3730	0.2736	0.2096	0.3288	0.3180	0.3622	0.3676	0.2268
-0.6428	**0.6878**	0.1864	0.6196	0.4194	0.3146	0.2362	0.3560	0.3614	0.3990	0.4052	0.2632
-0.6698	**0.7318**	0.2496	0.6816	0.4936	0.3648	0.2600	0.4044	0.4090	0.4476	0.4682	0.2936
-0.6948	**0.7672**	0.2778	0.7288	0.5356	0.4020	0.2910	0.4372	0.4376	0.4806	0.4964	0.3180
-0.7179	**0.8202**	0.3368	0.7884	0.5962	0.4550	0.3274	0.4830	0.4904	0.5354	0.5576	0.3624
-0.7394	**0.844**	0.3714	0.8218	0.6360	0.4696	0.3246	0.4916	0.5038	0.5462	0.5748	0.3700
-0.7593	**0.8736**	0.4300	0.8618	0.6922	0.5294	0.3696	0.5458	0.5588	0.6028	0.6334	0.3964
-0.7779	**0.8916**	0.4674	0.8810	0.7118	0.5510	0.3686	0.5486	0.5674	0.6124	0.6460	0.4224
-0.7953	0.9118	0.5372	**0.9144**	0.7678	0.5938	0.4102	0.5976	0.6148	0.6568	0.7004	0.4638
-0.8116	**0.9296**	0.5732	0.9264	0.7892	0.6290	0.4396	0.6244	0.6466	0.6888	0.7256	0.4826
-0.8268	**0.9398**	0.6116	0.9360	0.8200	0.6544	0.4518	0.6522	0.6690	0.7114	0.7502	0.5086
-0.8411	0.9446	0.6524	**0.9506**	0.8442	0.6802	0.4772	0.6664	0.6934	0.7358	0.7766	0.5354
-0.8546	0.9498	0.6934	**0.9614**	0.8744	0.7188	0.4984	0.6912	0.7274	0.7654	0.8106	0.5574
-0.8672	0.9584	0.7190	**0.9694**	0.8814	0.7268	0.5002	0.7072	0.7322	0.7738	0.8220	0.5606
-0.8792	**0.9694**	0.7394	0.9758	0.9056	0.7526	0.5276	0.7286	0.7568	0.7910	0.8404	0.5862

**Table 6:** Comparison of the powers of the tests for normality for GG distribution and *N* = 1000. The following tests are taken under consideration: the new test proposed in this paper, Jarque-Bera (JB) test [5], D’Agostino-Pearson (DP) test [4], Shapiro-Wilk (SW) test [3], test based on the empirical characteristic function (CF) [22], Kolmogorov-Smirnov (KS) test [2], Kuiper test [6], Watson test [7], Cramer-von Mises (CvM) test [8], Anderson-Darling (AD) test [9] and *χ*^2^ goodness-of-fit test [1].

*kurtosis*	*new test*	*JB*	*DP*	*SW*	*CF*	*KS*	*Kuiper*	*Watson*	*CvM*	*AD*	*χ*^2^
0.0000	0.0458	0.0484	0.0516	0.0580	0.0302	0.0490	0.0502	**0.0534**	0.0532	0.0512	0.0530
-0.0934	**0.0882**	0.0490	0.0770	0.0640	0.0380	0.0654	0.0710	0.0684	0.0722	0.0716	0.0680
-0.1753	**0.2268**	0.0916	0.1658	0.1162	0.0916	0.0960	0.1306	0.1194	0.1356	0.1256	0.0904
-0.2475	**0.4240**	0.2116	0.3416	0.2386	0.1770	0.1632	0.2338	0.2290	0.2542	0.2528	0.1566
-0.3116	**0.6388**	0.3762	0.5438	0.4012	0.2970	0.2586	0.3716	0.3714	0.4094	0.4000	0.2642
-0.3688	**0.8042**	0.5778	0.7318	0.5826	0.4370	0.3556	0.5086	0.5242	0.5664	0.5774	0.3908
-0.4202	**0.9176**	0.7530	0.8702	0.7576	0.6104	0.4928	0.6576	0.6960	0.7292	0.7402	0.5318
-0.4666	**0.9674**	0.8804	0.9496	0.8756	0.7136	0.6050	0.7712	0.7960	0.8230	0.8474	0.6604
-0.5086	**0.9882**	0.9520	0.9794	0.9448	0.8226	0.7048	0.8518	0.8842	0.9078	0.9286	0.7722
-0.5468	**0.9992**	0.9836	0.9962	0.9798	0.8924	0.7878	0.9204	0.9418	0.9554	0.9664	0.8586
-0.5816	**0.9996**	0.9944	0.9992	0.9934	0.9384	0.8670	0.9574	0.9686	0.9770	0.9838	0.9152
-0.6135	**1.0000**	0.9984	0.9998	0.9974	0.9694	0.9124	0.9740	0.9858	0.9892	0.9938	0.9536
-0.6428	**1.0000**	0.9998	1.0000	0.9992	0.9848	0.9482	0.9900	0.9936	0.9960	0.9976	0.9776
-0.6698	**1.0000**	0.9998	1.0000	0.9998	0.9924	0.9650	0.9942	0.9980	0.9984	0.9994	0.9890
-0.6948	**1.0000**	1.0000	1.0000	1.0000	0.9968	0.9782	0.9970	0.9980	0.9984	0.9992	0.9942
-0.7179	**1.0000**	1.0000	1.0000	1.0000	0.9988	0.9904	0.9986	0.9992	0.9996	1.0000	0.9978
-0.7394	**1.0000**	1.0000	1.0000	1.0000	0.9994	0.9934	0.9992	1.0000	1.0000	1.0000	0.9982
-0.7593	**1.0000**	1.0000	1.0000	1.0000	0.9996	0.9958	0.9998	1.0000	1.0000	1.0000	0.9990
-0.7779	**1.0000**	1.0000	1.0000	1.0000	1.0000	0.9984	1.0000	1.0000	1.0000	1.0000	1.0000
-0.7953	**1.0000**	1.0000	1.0000	1.0000	1.0000	0.9994	1.0000	1.0000	1.0000	1.0000	1.0000
-0.8116	**1.0000**	1.0000	1.0000	1.0000	1.0000	0.9996	1.0000	1.0000	1.0000	1.0000	0.9998
-0.8268	**1.0000**	1.0000	1.0000	1.0000	1.0000	0.9994	1.0000	1.0000	1.0000	1.0000	1.0000
-0.8411	**1.0000**	1.0000	1.0000	1.0000	1.0000	0.9998	1.0000	1.0000	1.0000	1.0000	1.0000
-0.8546	**1.0000**	1.0000	1.0000	1.0000	1.0000	0.9996	1.0000	1.0000	1.0000	1.0000	1.0000
-0.8672	**1.0000**	1.0000	1.0000	1.0000	1.0000	1.0000	1.0000	1.0000	1.0000	1.0000	1.0000
-0.8792	**1.0000**	1.0000	1.0000	1.0000	1.0000	1.0000	1.0000	1.0000	1.0000	1.0000	1.0000

**Table 7:** Comparison of the powers of the tests for normality for MG distribution and *N* = 20. The following tests are taken under consideration: the new test proposed in this paper, Jarque-Bera (JB) test [5], D’Agostino-Pearson (DP) test [4], Shapiro-Wilk (SW) test [3], test based on the empirical characteristic function (CF) [22], Kolmogorov-Smirnov (KS) test [2], Kuiper test [6], Watson test [7], Cramer-von Mises (CvM) test [8], Anderson-Darling (AD) test [9] and *χ*^2^ goodness-of-fit test [1].

*kurtosis*	*new test*	*JB*	*DP*	*SW*	*CF*	*KS*	*Kuiper*	*Watson*	*CvM*	*AD*	*χ*^2^
-0.0380	0.0466	0.0488	0.0546	**0.0594**	0.0422	0.0500	0.0488	0.0480	0.0480	0.0482	0.0000
-0.0567	0.0462	0.0478	0.0522	**0.0638**	0.0524	0.0502	0.0526	0.0540	0.0526	0.0526	0.0000
-0.0800	0.0456	0.0438	0.0502	**0.0584**	0.0430	0.0474	0.0476	0.0498	0.0488	0.0466	0.0000
-0.1079	0.0436	0.0436	0.0492	**0.0600**	0.0464	0.0520	0.0502	0.0504	0.0512	0.0494	0.0000
-0.1401	0.0428	0.0368	0.0484	**0.0534**	0.0480	0.0464	0.0462	0.0446	0.0452	0.0448	0.0000
-0.1764	0.0412	0.0330	0.0392	0.0458	**0.0522**	0.0368	0.0438	0.0408	0.0380	0.0368	0.0000
-0.2163	0.0380	0.0302	0.0366	**0.0474**	0.0472	0.0418	0.0434	0.0430	0.0422	0.0422	0.0000
-0.2592	0.0438	0.0284	0.0366	0.0504	**0.0548**	0.0476	0.0468	0.0466	0.0458	0.0452	0.0000
-0.3046	0.0456	0.0306	0.0384	0.0510	**0.0594**	0.0456	0.0460	0.0484	0.0470	0.0464	0.0000
-0.3519	0.0462	0.0258	0.0342	0.0476	**0.0616**	0.0508	0.0502	0.0506	0.0488	0.0460	0.0000
-0.4005	0.0460	0.0258	0.0352	0.0470	**0.0634**	0.0484	0.0470	0.0490	0.0460	0.0452	0.0000
-0.4501	0.0482	0.0164	0.0302	0.0438	**0.0682**	0.0466	0.0484	0.0458	0.0438	0.0440	0.0000
-0.5000	0.0444	0.0182	0.0264	0.0426	**0.0670**	0.0528	0.0574	0.0546	0.0504	0.0454	0.0000
-0.5499	0.0478	0.0174	0.0344	0.0556	**0.0804**	0.0572	0.0630	0.0616	0.0582	0.0580	0.0000
-0.5995	0.0512	0.0114	0.0304	0.0564	**0.1014**	0.0614	0.0696	0.0660	0.0608	0.0604	0.0000
-0.6485	0.0596	0.0142	0.0370	0.0562	**0.1046**	0.0572	0.0668	0.0660	0.0590	0.0576	0.0000
-0.6966	0.0546	0.0102	0.0376	0.0666	**0.1166**	0.0616	0.0712	0.0752	0.0680	0.0692	0.0000
-0.7436	0.0656	0.0106	0.0414	0.0714	**0.1426**	0.0746	0.0916	0.0884	0.0822	0.0770	0.0000
-0.7894	0.0728	0.0086	0.0430	0.0720	**0.1454**	0.0698	0.0888	0.0920	0.0826	0.0822	0.0000
-0.8339	0.0712	0.0086	0.0506	0.0920	**0.1672**	0.0898	0.1114	0.1152	0.1038	0.1008	0.0000
-0.8769	0.0858	0.0090	0.0634	0.1132	**0.1942**	0.1048	0.1346	0.1404	0.1264	0.1258	0.0000
-0.9185	0.0910	0.0064	0.0646	0.1168	**0.2108**	0.1124	0.1452	0.1512	0.1348	0.1314	0.0000
-0.9586	0.0924	0.0082	0.0768	0.1290	**0.2352**	0.1320	0.1694	0.1710	0.1538	0.1524	0.0000

**Table 8:** Comparison of the powers of the tests for normality for MG distribution and *N* = 50. The following tests are taken under consideration: the new test proposed in this paper, Jarque-Bera (JB) test [5], D’Agostino-Pearson (DP) test [4], Shapiro-Wilk (SW) test [3], test based on the empirical characteristic function (CF) [22], Kolmogorov-Smirnov (KS) test [2], Kuiper test [6], Watson test [7], Cramer-von Mises (CvM) test [8], Anderson-Darling (AD) test [9] and *χ*^2^ goodness-of-fit test [1].

*kurtosis*	*new test*	*JB*	*DP*	*SW*	*CF*	*KS*	*Kuiper*	*Watson*	*CvM*	*AD*	*χ*^2^
-0.0380	0.0442	0.0438	0.0482	0.0492	0.0254	0.0446	0.0474	0.0424	0.0434	0.0396	**0.0646**
-0.0567	0.0480	0.0426	0.0512	0.0580	0.0248	0.0474	0.0484	0.0482	0.0494	0.0504	**0.0564**
-0.0800	0.0398	0.0346	0.0446	0.0508	0.0252	0.0486	0.0458	0.0422	0.0450	0.0434	**0.0602**
-0.1079	0.0394	0.0358	0.0444	0.0480	0.0232	0.0418	0.0392	0.0448	0.0422	0.0426	**0.0590**
-0.1401	0.0448	0.0308	0.0478	0.0546	0.0268	0.0456	0.0466	0.0448	0.0460	0.0458	**0.0668**
-0.1764	0.0418	0.0292	0.0420	0.0486	0.0274	0.0452	0.0476	0.0464	0.0466	0.0456	**0.0594**
-0.2163	0.0414	0.0236	0.0394	0.0452	0.0246	0.0460	0.0480	0.0440	0.0470	0.0442	**0.0602**
-0.2592	0.0400	0.0196	0.0406	0.0432	0.0256	0.0442	0.0488	0.0436	0.0460	0.0430	**0.0600**
-0.3046	0.0398	0.0182	0.0412	0.0506	0.0304	0.0508	0.0534	0.0492	0.0526	0.0504	**0.063**
-0.3519	0.0506	0.0152	0.0512	0.0522	0.0388	0.0558	0.0650	0.0600	**0.0648**	0.0570	0.0642
-0.4005	0.0546	0.0138	0.0586	0.0584	0.0440	0.0584	0.0662	0.0596	0.0666	0.0608	**0.069**
-0.4501	0.0556	0.0090	0.0616	0.0598	0.0524	0.0626	**0.0802**	0.0724	0.0794	0.0742	0.0764
-0.5000	0.0612	0.0084	0.0732	0.0682	0.0584	0.0726	**0.0868**	0.0812	0.0872	0.0780	0.0814
-0.5499	0.0674	0.0050	0.0834	0.0758	0.0752	0.0792	0.0970	0.0902	**0.1018**	0.0942	0.0888
-0.5995	0.0704	0.0062	0.1118	0.0942	0.0924	0.0888	0.1150	0.1080	**0.1190**	0.1106	0.0920
-0.6485	0.0890	0.0050	0.1336	0.1104	0.1178	0.1002	0.1428	0.1302	**0.1446**	0.1316	0.0988
-0.6966	0.0894	0.0036	0.1500	0.1194	0.1376	0.1098	0.1570	0.1436	**0.1610**	0.1464	0.1044
-0.7436	0.1154	0.0034	0.1986	0.1634	0.1884	0.1466	0.2056	0.1874	**0.2088**	0.1884	0.1288
-0.7894	0.1234	0.0036	0.2374	0.1900	0.2254	0.1646	0.2274	0.2206	**0.2466**	0.2292	0.1478
-0.8339	0.1434	0.0028	0.2678	0.2236	0.2702	0.1932	0.2818	0.2676	**0.2978**	0.2668	0.1688
-0.8769	0.1520	0.0032	0.3376	0.2868	0.3448	0.2416	0.3406	0.3338	**0.3678**	0.3354	0.2028
-0.9185	0.1876	0.0012	0.3644	0.3138	0.3764	0.2720	0.3802	0.3644	**0.4000**	0.3672	0.2196
-0.9586	0.1912	0.0026	0.4304	0.3750	0.4480	0.3176	0.4350	0.4320	**0.4748**	0.4368	0.2520

**Table 9:** Comparison of the powers of the tests for normality for MG distribution and *N* = 100. The following tests are taken under consideration: the new test proposed in this paper, Jarque-Bera (JB) test [5], D’Agostino-Pearson (DP) test [4], Shapiro-Wilk (SW) test [3], test based on the empirical characteristic function (CF) [22], Kolmogorov-Smirnov (KS) test [2], Kuiper test [6], Watson test [7], Cramer-von Mises (CvM) test [8], Anderson-Darling (AD) test [9] and *χ*^2^ goodness-of-fit test [1].

*kurtosis*	*new test*	*JB*	*DP*	*SW*	*CF*	*KS*	*Kuiper*	*Watson*	*CvM*	*AD*	*χ*^2^
-0.0380	0.0460	0.0390	0.0442	0.0548	0.0202	0.0534	0.0516	0.0470	0.0460	0.0476	**0.0554**
-0.0567	0.0436	0.0366	0.0458	**0.0506**	0.0190	0.0484	0.0466	0.0462	0.0470	0.0462	0.0484
-0.0800	0.0536	0.0368	0.0480	0.0482	0.0222	0.0502	0.0496	0.0448	0.0444	0.0444	**0.0552**
-0.1079	0.0408	0.0310	0.0410	0.0466	0.0198	0.0442	0.0428	0.0440	0.0446	0.0430	**0.0572**
-0.1401	0.0544	0.0288	0.0522	0.0564	0.0226	0.0492	0.0494	0.0480	0.0496	0.0472	**0.0606**
-0.1764	0.0586	0.0242	0.0480	0.0470	0.0228	0.0420	0.0502	0.0470	0.0500	0.0458	**0.0624**
-0.2163	**0.0626**	0.0170	0.0474	0.0450	0.0208	0.0462	0.0506	0.0502	0.0530	0.0518	0.0592
-0.2592	**0.0720**	0.0156	0.0576	0.0492	0.0258	0.0504	0.0578	0.0522	0.0574	0.0556	0.0614
-0.3046	**0.0860**	0.0120	0.0632	0.0570	0.0330	0.0544	0.0672	0.0660	0.0700	0.0650	0.0732
-0.3519	**0.1056**	0.0122	0.0820	0.0686	0.0428	0.0688	0.0874	0.0792	0.0878	0.0844	0.0800
-0.4005	**0.1300**	0.0108	0.1034	0.0830	0.0536	0.0764	0.0978	0.0880	0.0968	0.0924	0.0862
-0.4501	**0.1622**	0.0088	0.1260	0.0868	0.0648	0.0746	0.1040	0.0998	0.1084	0.1048	0.0838
-0.5000	**0.1940**	0.0066	0.1578	0.1098	0.0948	0.0994	0.1316	0.1312	0.1442	0.1362	0.1190
-0.5499	**0.2394**	0.0112	0.2100	0.1430	0.1192	0.1246	0.1704	0.1634	0.1816	0.1724	0.1366
-0.5995	**0.2840**	0.0116	0.2466	0.1808	0.1682	0.1486	0.2162	0.2092	0.2330	0.2202	0.1500
-0.6485	**0.3468**	0.0172	0.3272	0.2296	0.2268	0.1794	0.2632	0.2592	0.2872	0.2706	0.1868
-0.6966	**0.3948**	0.0276	0.3898	0.2786	0.2838	0.2234	0.3234	0.3226	0.3588	0.3332	0.2162
-0.7436	0.4790	0.0400	**0.4858**	0.3632	0.3790	0.2786	0.4076	0.4124	0.4546	0.4270	0.2658
-0.7894	0.5284	0.0606	**0.5494**	0.4180	0.4548	0.3260	0.4672	0.4750	0.5216	0.4920	0.3214
-0.8339	0.5852	0.0834	**0.6248**	0.5040	0.5444	0.4132	0.5602	0.5716	0.6110	0.5842	0.3798
-0.8769	0.6532	0.1220	**0.7046**	0.6038	0.6426	0.4884	0.6510	0.6674	0.7034	0.6826	0.4604
-0.9185	0.7030	0.1626	0.7726	0.6778	0.7152	0.5644	0.7186	0.7392	**0.7728**	0.7500	0.5332
-0.9586	0.7542	0.2144	0.8298	0.7434	0.7890	0.6278	0.7798	0.8060	**0.8316**	0.8122	0.5828

**Table 10:** Comparison of the powers of the tests for normality for MG distribution and *N* = 200. The following tests are taken under consideration: the new test proposed in this paper, Jarque-Bera (JB) test [5], D’Agostino-Pearson (DP) test [4], Shapiro-Wilk (SW) test [3], test based on the empirical characteristic function (CF) [22], Kolmogorov-Smirnov (KS) test [2], Kuiper test [6], Watson test [7], Cramer-von Mises (CvM) test [8], Anderson-Darling (AD) test [9] and *χ*^2^ goodness-of-fit test [1].

*kurtosis*	*new test*	*JB*	*DP*	*SW*	*CF*	*KS*	*Kuiper*	*Watson*	*CvM*	*AD*	*χ*^2^
-0.0380	0.0464	0.0378	0.0454	0.0514	0.0224	**0.0546**	0.0512	0.0494	0.0494	0.0478	0.0534
-0.0567	0.0490	0.0348	0.0476	**0.0556**	0.0226	0.0542	0.0500	0.0512	0.0504	0.0508	0.0498
-0.0800	0.0492	0.0322	0.0494	**0.0506**	0.0222	0.0462	0.0424	0.0446	0.0456	0.0464	0.0498
-0.1079	**0.0546**	0.0248	0.0450	0.0452	0.0216	0.0444	0.0472	0.0408	0.0434	0.0426	0.0536
-0.1401	**0.0638**	0.0294	0.0562	0.0568	0.0268	0.0542	0.0574	0.0574	0.0594	0.0580	0.0602
-0.1764	**0.0730**	0.0206	0.0558	0.0502	0.0236	0.0508	0.0614	0.0554	0.0578	0.0550	0.0628
-0.2163	**0.0996**	0.0192	0.0718	0.0524	0.0264	0.0534	0.0592	0.0558	0.0602	0.0570	0.0700
-0.2592	**0.1232**	0.0202	0.0852	0.0652	0.0354	0.0600	0.0736	0.0680	0.0728	0.0694	0.0746
-0.3046	**0.1592**	0.0240	0.1108	0.0854	0.0564	0.0778	0.0954	0.0954	0.1072	0.0978	0.0848
-0.3519	**0.2042**	0.0248	0.1496	0.1062	0.0726	0.0906	0.1228	0.1134	0.1258	0.1206	0.0974
-0.4005	**0.2576**	0.0370	0.2008	0.1382	0.1094	0.1176	0.1582	0.1518	0.1708	0.1570	0.1220
-0.4501	**0.3454**	0.0538	0.2724	0.1726	0.1466	0.1320	0.1964	0.1840	0.2100	0.1996	0.1438
-0.5000	**0.4164**	0.0658	0.3470	0.2350	0.2054	0.1748	0.2536	0.2562	0.2848	0.2718	0.1842
-0.5499	**0.5082**	0.1198	0.4400	0.3118	0.2960	0.2292	0.3348	0.3390	0.3756	0.3612	0.2476
-0.5995	**0.5828**	0.1612	0.5322	0.3968	0.3796	0.2934	0.4248	0.4192	0.4638	0.4446	0.2998
-0.6485	**0.6896**	0.2460	0.6528	0.5148	0.5074	0.3846	0.5386	0.5422	0.5850	0.5730	0.3832
-0.6966	**0.7692**	0.3262	0.7354	0.6038	0.6160	0.4698	0.6272	0.6480	0.6868	0.6686	0.4570
-0.7436	**0.8338**	0.4410	0.8250	0.7222	0.7350	0.5714	0.7408	0.7626	0.7994	0.7816	0.5608
-0.7894	0.8800	0.5306	**0.8872**	0.8080	0.8314	0.6690	0.8266	0.8502	0.8770	0.8654	0.6568
-0.8339	0.9150	0.6442	**0.932**	0.8726	0.8976	0.7598	0.8894	0.9082	0.9252	0.9184	0.7432
-0.8769	0.9444	0.7570	0.9600	0.9326	0.9476	0.8448	0.9396	0.9522	**0.9636**	0.9556	0.8246
-0.9185	0.9632	0.8216	0.9784	0.9596	0.9712	0.9000	0.9648	0.9738	**0.9796**	0.9788	0.8828
-0.9586	0.9734	0.8944	0.9880	0.9824	0.9852	0.9434	0.9818	0.9876	**0.9912**	0.9902	0.9312

**Table 11:** Comparison of the powers of the tests for normality for MG distribution and *N* = 1000. The following tests are taken under consideration: the new test proposed in this paper, Jarque-Bera (JB) test [5], D’Agostino-Pearson (DP) test [4], Shapiro-Wilk (SW) test [3], test based on the empirical characteristic function (CF) [22], Kolmogorov-Smirnov (KS) test [2], Kuiper test [6], Watson test [7], Cramer-von Mises (CvM) test [8], Anderson-Darling (AD) test [9] and *χ*^2^ goodness-of-fit test [1].

*kurtosis*	*new test*	*JB*	*DP*	*SW*	*CF*	*KS*	*Kuiper*	*Watson*	*CvM*	*AD*	*χ*^2^
-0.0380	0.0540	0.0332	0.0446	0.0496	0.0298	**0.0554**	0.0524	0.0538	0.0536	0.0528	0.0496
-0.0567	**0.0650**	0.0394	0.0588	0.0598	0.0290	0.0478	0.0474	0.0488	0.0508	0.0476	0.0576
-0.0800	**0.0774**	0.0398	0.0646	0.0596	0.0264	0.0484	0.0508	0.0538	0.0538	0.0572	0.0554
-0.1079	**0.1010**	0.0448	0.0794	0.0640	0.0324	0.0576	0.0634	0.0614	0.0630	0.0656	0.0580
-0.1401	**0.1406**	0.0546	0.1054	0.0826	0.0524	0.0640	0.0840	0.0776	0.0848	0.0816	0.0686
-0.1764	**0.2216**	0.0906	0.1662	0.1132	0.0728	0.0832	0.1130	0.1060	0.1214	0.1196	0.0908
-0.2163	**0.3260**	0.1538	0.2616	0.1766	0.1116	0.1194	0.1630	0.1530	0.1730	0.1748	0.1242
-0.2592	**0.4624**	0.2384	0.3742	0.2588	0.1780	0.1624	0.2318	0.2322	0.2576	0.2630	0.1704
-0.3046	**0.6178**	0.3692	0.5324	0.3940	0.3026	0.2510	0.3646	0.3766	0.4082	0.4092	0.2604
-0.3519	**0.7558**	0.5266	0.6842	0.5418	0.4382	0.3614	0.5110	0.5148	0.5550	0.5634	0.3726
-0.4005	**0.8812**	0.6916	0.8276	0.7134	0.6130	0.4956	0.6504	0.6822	0.7206	0.7286	0.5182
-0.4501	**0.9498**	0.8424	0.9224	0.8628	0.7954	0.6666	0.8156	0.8440	0.8714	0.8782	0.6788
-0.5000	**0.9824**	0.9294	0.9692	0.9438	0.9050	0.8030	0.9136	0.9372	0.9476	0.9538	0.8274
-0.5499	**0.9952**	0.9760	0.9928	0.9860	0.9772	0.9168	0.9748	0.9852	0.9888	0.9894	0.9246
-0.5995	0.9988	0.9950	0.999	0.9974	0.9958	0.9708	0.9964	0.9988	0.9994	**0.9996**	0.9756
-0.6485	**1.0000**	0.9996	1.0000	0.9998	0.9996	0.9938	0.9992	0.9998	1.0000	1.0000	0.9968
-0.6966	**1.0000**	1.0000	1.0000	1.0000	0.9998	1.0000	1.0000	1.0000	1.0000	1.0000	0.9994
-0.7436	**1.0000**	1.0000	1.0000	1.0000	1.0000	0.9998	1.0000	1.0000	1.0000	1.0000	1.0000
-0.7894	**1.0000**	1.0000	1.0000	1.0000	1.0000	1.0000	1.0000	1.0000	1.0000	1.0000	1.0000
-0.8339	**1.0000**	1.0000	1.0000	1.0000	1.0000	1.0000	1.0000	1.0000	1.0000	1.0000	1.0000
-0.8769	**1.0000**	1.0000	1.0000	1.0000	1.0000	1.0000	1.0000	1.0000	1.0000	1.0000	1.0000
-0.9185	**1.0000**	1.0000	1.0000	1.0000	1.0000	1.0000	1.0000	1.0000	1.0000	1.0000	1.0000
-0.9586	**1.0000**	1.0000	1.0000	1.0000	1.0000	1.0000	1.0000	1.0000	1.0000	1.0000	1.0000

**Table 12:** Comparison of the powers of the tests for normality for *α*-stable distribution and *N* = 20. The following tests are taken under consideration: the new test proposed in this paper, Jarque-Bera (JB) test [5], D’Agostino-Pearson (DP) test [4], Shapiro-Wilk (SW) test [3], test based on the empirical characteristic function (CF) [22], Kolmogorov-Smirnov (KS) test [2], Kuiper test [6], Watson test [7], Cramer-von Mises (CvM) test [8], Anderson-Darling (AD) test [9] and *χ*^2^ goodness-of-fit test [1].

*α*	*new test*	*JB*	*DP*	*SW*	*CF*	*KS*	*Kuiper*	*Watson*	*CvM*	*AD*	*χ*^2^
2	0.0462	0.0472	0.0574	**0.0654**	0.0508	0.0456	0.0476	0.0484	0.0468	0.0450	0.0000
1.99	0.0518	0.0536	0.0710	**0.0762**	0.0572	0.0498	0.0506	0.0506	0.0498	0.0506	0.0000
1.98	0.0608	0.0716	0.0760	**0.0840**	0.0586	0.0554	0.0570	0.0600	0.0614	0.0612	0.0000
1.97	0.0822	0.0876	0.0892	**0.0940**	0.0680	0.0776	0.0722	0.0710	0.0740	0.0758	0.0000
1.96	0.0756	0.0864	0.0984	**0.1014**	0.0754	0.0736	0.0748	0.0758	0.0784	0.0846	0.0000

**Table 13:** Comparison of the powers of the tests for normality for *α*-stable distribution and *N* = 50. The following tests are taken under consideration: the new test proposed in this paper, Jarque-Bera (JB) test [5], D’Agostino-Pearson (DP) test [4], Shapiro-Wilk (SW) test [3], test based on the empirical characteristic function (CF) [22], Kolmogorov-Smirnov (KS) test [2], Kuiper test [6], Watson test [7], Cramer-von Mises (CvM) test [8], Anderson-Darling (AD) test [9] and *χ*^2^ goodness-of-fit test [1].

*α*	*new test*	*JB*	*DP*	*SW*	*CF*	*KS*	*Kuiper*	*Watson*	*CvM*	*AD*	*χ*^2^
2	0.0446	0.0454	0.0502	0.0570	0.0252	0.0464	0.0468	0.0434	0.0440	0.0430	**0.0576**
1.99	0.0678	0.0740	0.0754	**0.0798**	0.0356	0.0628	0.0626	0.0634	0.0608	0.0650	0.0660
1.98	0.0854	0.0988	0.1024	**0.1036**	0.0426	0.0624	0.0644	0.0710	0.0670	0.0754	0.0790
1.97	0.1128	0.1298	0.1344	**0.1376**	0.0548	0.0788	0.0828	0.0878	0.0844	0.0962	0.0876
1.96	0.1340	0.1608	0.1602	**0.1554**	0.0630	0.0978	0.0958	0.0968	0.0948	0.1050	0.0992

**Table 14:** Comparison of the powers of the tests for normality for *α*-stable distribution and *N* = 100. The following tests are taken under consideration: the new test proposed in this paper, Jarque-Bera (JB) test [5], D’Agostino-Pearson (DP) test [4], Shapiro-Wilk (SW) test [3], test based on the empirical characteristic function (CF) [22], Kolmogorov-Smirnov (KS) test [2], Kuiper test [6], Watson test [7], Cramer-von Mises (CvM) test [8], Anderson-Darling (AD) test [9] and *χ*^2^ goodness-of-fit test [1].

*α*	*new test*	*JB*	*DP*	*SW*	*CF*	*KS*	*Kuiper*	*Watson*	*CvM*	*AD*	*χ*^2^
2	0.0460	0.0486	0.0532	**0.0594**	0.0228	0.0518	0.0502	0.0506	0.0504	0.0464	0.0580
1.99	0.0876	0.0944	0.0932	**0.1020**	0.0356	0.0644	0.0672	0.0682	0.0706	0.0748	0.0686
1.98	0.1276	0.1360	0.1368	**0.1452**	0.0498	0.0814	0.0852	0.0862	0.0874	0.0936	0.0750
1.97	0.1752	**0.1922**	0.1894	0.1884	0.0662	0.0928	0.1000	0.1094	0.1066	0.1216	0.0898
1.96	0.2026	**0.2280**	0.2174	0.2254	0.0808	0.1090	0.1200	0.1246	0.1218	0.1362	0.1042

**Table 15:** Comparison of the powers of the tests for normality for *α*-stable distribution and *N* = 200. The following tests are taken under consideration: the new test proposed in this paper, Jarque-Bera (JB) test [5], D’Agostino-Pearson (DP) test [4], Shapiro-Wilk (SW) test [3], test based on the empirical characteristic function (CF) [22], Kolmogorov-Smirnov (KS) test [2], Kuiper test [6], Watson test [7], Cramer-von Mises (CvM) test [8], Anderson-Darling (AD) test [9] and *χ*^2^ goodness-of-fit test [1].

*α*	*new test*	*JB*	*DP*	*SW*	*CF*	*KS*	*Kuiper*	*Watson*	*CvM*	*AD*	*χ*^2^
2	0.0532	0.0538	0.0582	**0.0652**	0.0244	0.0510	0.0536	0.0492	0.0484	0.0498	0.0536
1.99	0.1198	0.1342	0.1278	**0.1390**	0.0448	0.0656	0.0692	0.0728	0.0738	0.0792	0.0672
1.98	0.1836	0.2016	0.1978	**0.2084**	0.0700	0.0954	0.1050	0.1134	0.1132	0.1246	0.0810
1.97	0.2604	**0.2882**	0.2776	0.2810	0.0950	0.1188	0.1328	0.1388	0.1406	0.1570	0.1070
1.96	0.3136	**0.3410**	0.3280	0.3338	0.1148	0.1442	0.1558	0.1658	0.1650	0.1888	0.1168

**Table 16:** Comparison of the powers of the tests for normality for *α*-stable distribution and *N* = 1000. The following tests are taken under consideration: the new test proposed in this paper, Jarque-Bera (JB) test [5], D’Agostino-Pearson (DP) test [4], Shapiro-Wilk (SW) test [3], test based on the empirical characteristic function (CF) [22], Kolmogorov-Smirnov (KS) test [2], Kuiper test [6], Watson test [7], Cramer-von Mises (CvM) test [8], Anderson-Darling (AD) test [9] and *χ*^2^ goodness-of-fit test [1].

*α*	*new test*	*JB*	*DP*	*SW*	*CF*	*KS*	*Kuiper*	*Watson*	*CvM*	*AD*	*χ*^2^
2	0.0504	0.0530	0.0514	**0.0618**	0.0250	0.0520	0.0480	0.0548	0.0528	0.0544	0.0466
1.99	0.3014	0.3124	0.3002	**0.3232**	0.0758	0.1010	0.1138	0.1210	0.1184	0.1440	0.0884
1.98	0.5310	0.5364	0.5176	**0.5448**	0.1426	0.1664	0.1980	0.2052	0.2076	0.2480	0.1392
1.97	**0.6926**	0.6924	0.6704	0.6920	0.2218	0.2406	0.2948	0.3062	0.3096	0.3674	0.1882
1.96	**0.7862**	0.7862	0.7662	0.7840	0.3004	0.3192	0.3820	0.3940	0.4008	0.4736	0.2440

**Table 17:** Comparison of the powers of the tests for normality for Student’s t distribution and *N* = 20. The following tests are taken under consideration: the new test proposed in this paper, Jarque-Bera (JB) test [5], D’Agostino-Pearson (DP) test [4], Shapiro-Wilk (SW) test [3], test based on the empirical characteristic function (CF) [22], Kolmogorov-Smirnov (KS) test [2], Kuiper test [6], Watson test [7], Cramer-von Mises (CvM) test [8], Anderson-Darling (AD) test [9] and *χ*^2^ goodness-of-fit test [1].

*ν*	*new test*	*JB*	*DP*	*SW*	*CF*	*KS*	*Kuiper*	*Watson*	*CvM*	*AD*	*χ*^2^
36	0.0582	0.0660	0.0696	**0.0774**	0.0512	0.0474	0.0530	0.0564	0.0576	0.0578	0.0000
34	0.0520	0.0626	**0.0670**	0.0674	0.0436	0.0458	0.0456	0.0494	0.0498	0.0502	0.0000
30	0.0526	0.0680	0.0722	**0.0756**	0.0524	0.0552	0.0588	0.0586	0.0588	0.0602	0.0000
26	0.0578	0.0776	**0.0822**	0.0806	0.0540	0.0584	0.0566	0.0546	0.0556	0.0580	0.0000
22	0.0614	0.0844	**0.0906**	0.0878	0.0652	0.0612	0.0608	0.0606	0.0608	0.0664	0.0000
20	0.0630	0.0796	0.0852	**0.0886**	0.0562	0.0592	0.0582	0.0596	0.0608	0.0634	0.0000
18	0.0582	0.0790	**0.0838**	0.0826	0.0538	0.0530	0.0518	0.0560	0.0596	0.0614	0.0000
16	0.0660	0.0872	**0.0914**	0.0876	0.0596	0.0614	0.0584	0.0614	0.0614	0.0630	0.0000
14	0.0698	0.0968	**0.1016**	0.1004	0.0630	0.0612	0.0604	0.0640	0.0686	0.0736	0.0000
12	0.0764	0.1142	**0.1186**	0.1140	0.0676	0.0660	0.0642	0.0678	0.0726	0.0772	0.0000
10	0.0946	0.1348	**0.1392**	0.1298	0.0776	0.0734	0.0774	0.0826	0.0880	0.0980	0.0000
8	0.1044	0.1496	**0.1516**	0.1496	0.0944	0.0838	0.0866	0.0942	0.0944	0.1036	0.0000
6	0.1430	0.1970	**0.2002**	0.1974	0.1258	0.1052	0.1164	0.1312	0.1342	0.1466	0.0000
4	0.2070	0.2928	0.2926	**0.2928**	0.2018	0.1746	0.1860	0.1964	0.2072	0.2224	0.0000
2	0.4696	0.5728	0.5674	**0.5898**	0.5096	0.4594	0.4848	0.5062	0.5138	0.5312	0.0000

**Table 18:** Comparison of the powers of the tests for normality for Student’s t distribution and *N* = 50. The following tests are taken under consideration: the new test proposed in this paper, Jarque-Bera (JB) test [5], D’Agostino-Pearson (DP) test [4], Shapiro-Wilk (SW) test [3], test based on the empirical characteristic function (CF) [22], Kolmogorov-Smirnov (KS) test [2], Kuiper test [6], Watson test [7], Cramer-von Mises (CvM) test [8], Anderson-Darling (AD) test [9] and *χ*^2^ goodness-of-fit test [1].

*ν*	*new test*	*JB*	*DP*	*SW*	*CF*	*KS*	*Kuiper*	*Watson*	*CvM*	*AD*	*χ*^2^
36	0.0672	0.0846	0.0866	**0.0946**	0.0340	0.0538	0.0580	0.0574	0.0578	0.0608	0.0694
34	0.0556	0.0744	0.0756	**0.0790**	0.0256	0.0490	0.0464	0.0498	0.0448	0.0522	0.0588
30	0.0650	0.0942	0.0924	**0.0982**	0.0324	0.0530	0.0494	0.0548	0.0534	0.0568	0.0602
26	0.0718	0.1016	0.0970	**0.0990**	0.0330	0.0550	0.0562	0.0596	0.0590	0.0610	0.0658
22	0.0770	0.1082	0.1028	**0.1096**	0.0346	0.0576	0.0588	0.0626	0.0606	0.0660	0.0674
20	0.0832	0.1080	0.1008	**0.1102**	0.0380	0.0610	0.0594	0.0626	0.0608	0.0664	0.0760
18	0.0874	0.1206	0.1174	**0.1212**	0.0444	0.0586	0.0628	0.0718	0.0680	0.0770	0.0672
16	0.0920	**0.1318**	0.1244	0.1262	0.0438	0.0682	0.0676	0.0700	0.0686	0.0796	0.0718
14	0.1098	**0.1532**	0.1430	0.1478	0.0542	0.0722	0.0748	0.0818	0.0810	0.0932	0.0804
12	0.1270	**0.1800**	0.1714	0.1764	0.0580	0.0742	0.0814	0.0896	0.0900	0.1018	0.0806
10	0.1568	**0.2200**	0.2040	0.2042	0.0756	0.0904	0.0990	0.1090	0.1064	0.1242	0.0872
8	0.2016	**0.2620**	0.2450	0.2574	0.1056	0.1166	0.1294	0.1472	0.1420	0.1692	0.1086
6	0.2718	**0.3448**	0.3174	0.3354	0.1582	0.1538	0.1856	0.2008	0.1998	0.2258	0.1396
4	0.4574	**0.5418**	0.5134	0.5300	0.3288	0.3048	0.3464	0.3882	0.3794	0.4174	0.2674
2	0.8378	0.8850	0.8632	**0.8952**	0.8256	0.7762	0.8182	0.8398	0.8446	0.8560	0.7054

**Table 19:** Comparison of the powers of the tests for normality for Student’s t distribution and *N* = 100. The following tests are taken under consideration: the new test proposed in this paper, Jarque-Bera (JB) test [5], D’Agostino-Pearson (DP) test [4], Shapiro-Wilk (SW) test [3], test based on the empirical characteristic function (CF) [22], Kolmogorov-Smirnov (KS) test [2], Kuiper test [6], Watson test [7], Cramer-von Mises (CvM) test [8], Anderson-Darling (AD) test [9] and *χ*^2^ goodness-of-fit test [1].

*ν*	*new test*	*JB*	*DP*	*SW*	*CF*	*KS*	*Kuiper*	*Watson*	*CvM*	*AD*	*χ*^2^
36	0.0780	0.0976	0.0902	**0.0984**	0.0538	0.0348	0.0604	0.0598	0.0606	0.0642	0.0624
34	0.0748	0.0934	0.0864	**0.0944**	0.0520	0.0298	0.0522	0.0518	0.0506	0.0560	0.0556
30	0.0922	0.1074	0.1022	**0.1116**	0.0596	0.0372	0.0632	0.0640	0.0618	0.0658	0.0600
26	0.0954	**0.1216**	0.1070	0.1158	0.0544	0.0386	0.0578	0.0594	0.0610	0.0652	0.0594
22	0.1096	**0.1388**	0.1262	0.1342	0.0654	0.0434	0.0702	0.0752	0.0736	0.0782	0.0558
20	0.1096	**0.1438**	0.1288	0.1342	0.0614	0.0372	0.0682	0.0680	0.0662	0.0754	0.0590
18	0.1226	**0.1638**	0.1458	0.1636	0.0692	0.0472	0.0752	0.0808	0.0806	0.0878	0.0630
16	0.1388	**0.1824**	0.1634	0.1714	0.0716	0.0550	0.0816	0.0828	0.0850	0.0920	0.0638
14	0.1646	**0.2174**	0.1932	0.1994	0.0756	0.0628	0.0830	0.0946	0.0922	0.1056	0.0702
12	0.1978	**0.2554**	0.2268	0.2402	0.0908	0.0716	0.1058	0.1092	0.1086	0.1256	0.0744
10	0.2626	**0.3244**	0.2878	0.3026	0.1114	0.1078	0.1358	0.1456	0.1470	0.1756	0.0918
8	0.3376	**0.4076**	0.3678	0.3862	0.1496	0.1516	0.1840	0.2058	0.2082	0.2458	0.1122
6	0.4680	**0.5294**	0.4848	0.5096	0.2352	0.2536	0.2898	0.3092	0.3108	0.3502	0.1690
4	0.7236	**0.7694**	0.7258	0.7590	0.4812	0.5492	0.5666	0.5944	0.6050	0.6408	0.3910
2	0.9834	0.9892	0.9814	**0.9914**	0.9568	0.9780	0.9750	0.9822	0.9832	0.9862	0.8806

**Table 20:** Comparison of the powers of the tests for normality for Student’s t distribution and *N* = 200. The following tests are taken under consideration: the new test proposed in this paper, Jarque-Bera (JB) test [5], D’Agostino-Pearson (DP) test [4], Shapiro-Wilk (SW) test [3], test based on the empirical characteristic function (CF) [22], Kolmogorov-Smirnov (KS) test [2], Kuiper test [6], Watson test [7], Cramer-von Mises (CvM) test [8], Anderson-Darling (AD) test [9] and *χ*^2^ goodness-of-fit test [1].

*ν*	*new test*	*JB*	*DP*	*SW*	*CF*	*KS*	*Kuiper*	*Watson*	*CvM*	*AD*	*χ*^2^
36	0.0908	**0.1278**	0.1122	0.1196	0.0400	0.0640	0.0638	0.0680	0.0664	0.0708	0.0558
34	0.0840	**0.1210**	0.1048	0.1162	0.0402	0.0568	0.0596	0.0584	0.0594	0.0594	0.0514
30	0.1020	**0.1362**	0.1154	0.1322	0.0428	0.0584	0.0672	0.0690	0.0702	0.0736	0.0528
26	0.1142	**0.1584**	0.1346	0.1496	0.0466	0.0656	0.0738	0.0748	0.0750	0.0776	0.0586
22	0.1382	**0.1920**	0.1644	0.1740	0.0536	0.0682	0.0824	0.0782	0.0780	0.0884	0.0592
20	0.1504	**0.2056**	0.1762	0.1908	0.0564	0.0762	0.0860	0.0934	0.0898	0.1058	0.0666
18	0.1716	**0.2224**	0.1886	0.2042	0.0648	0.0744	0.0874	0.0916	0.0924	0.1000	0.0630
16	0.2036	**0.2672**	0.2202	0.2384	0.0742	0.0898	0.1000	0.1052	0.1058	0.1194	0.0648
14	0.2520	**0.3154**	0.2736	0.2872	0.0898	0.0874	0.1184	0.1214	0.1240	0.1428	0.0738
12	0.3050	**0.3746**	0.3264	0.3448	0.1156	0.1124	0.1420	0.1524	0.1594	0.1788	0.0880
10	0.4088	**0.4760**	0.4214	0.4450	0.1730	0.1586	0.2050	0.2174	0.2210	0.2592	0.1132
8	0.5438	**0.6006**	0.5388	0.5632	0.2612	0.2254	0.2912	0.3116	0.3178	0.3644	0.1606
6	0.7216	**0.7670**	0.7170	0.7454	0.4674	0.3802	0.4794	0.5262	0.5342	0.5828	0.2886
4	0.9340	**0.9480**	0.9266	0.9420	0.8294	0.7372	0.8258	0.8504	0.8612	0.8814	0.6438
2	**0.9996**	0.9996	0.9996	0.9996	0.9996	0.9986	0.9994	0.9994	0.9996	0.9996	0.8828

**Table 21:** Comparison of the powers of the tests for normality for Student’s t distribution and *N* = 1000. The following tests are taken under consideration: the new test proposed in this paper, Jarque-Bera (JB) test [5], D’Agostino-Pearson (DP) test [4], Shapiro-Wilk (SW) test [3], test based on the empirical characteristic function (CF) [22], Kolmogorov-Smirnov (KS) test [2], Kuiper test [6], Watson test [7], Cramer-von Mises (CvM) test [8], Anderson-Darling (AD) test [9] and *χ*^2^ goodness-of-fit test [1].

*ν*	*new test*	*JB*	*DP*	*SW*	*CF*	*KS*	*Kuiper*	*Watson*	*CvM*	*AD*	*χ*^2^
36	0.2360	**0.2378**	0.1970	0.2140	0.0628	0.0682	0.0862	0.0890	0.0954	0.0998	0.0760
34	0.2380	**0.2458**	0.2050	0.2190	0.0590	0.0772	0.0988	0.0964	0.1014	0.1088	0.0868
30	0.2890	**0.2894**	0.2150	0.2560	0.0728	0.0874	0.1048	0.1058	0.1154	0.1224	0.0868
26	0.3558	**0.3568**	0.3080	0.3190	0.0838	0.0948	0.1206	0.1280	0.1344	0.1430	0.0994
22	**0.4568**	0.4428	0.3830	0.3940	0.1138	0.1132	0.1572	0.1644	0.1734	0.1974	0.1260
20	**0.5086**	0.4942	0.4370	0.4530	0.1348	0.1348	0.1866	0.1874	0.2004	0.2276	0.1552
18	**0.5972**	0.5798	0.5150	0.5250	0.1624	0.1626	0.2284	0.2328	0.2480	0.2804	0.1776
16	**0.6660**	0.6464	0.5900	0.6020	0.2120	0.1958	0.2806	0.2910	0.3118	0.3528	0.2100
14	**0.7772**	0.7646	0.7080	0.7150	0.2938	0.2564	0.3674	0.3874	0.4072	0.4668	0.2762
12	**0.8710**	0.8568	0.8180	0.8280	0.4094	0.3502	0.4822	0.5050	0.5376	0.5954	0.3692
10	**0.9462**	0.9380	0.9020	0.9250	0.6206	0.5192	0.6850	0.7128	0.7364	0.7874	0.5230
8	**0.9934**	0.9912	0.9860	0.9880	0.8498	0.7542	0.8758	0.8994	0.9112	0.9394	0.7386
6	**0.9998**	0.9998	0.9991	1.0000	0.9896	0.9640	0.9874	0.9936	0.9948	0.9974	0.9466
4	**1.0000**	1.0000	1.0000	1.0000	1.0000	0.9998	1.0000	1.0000	1.0000	1.0000	0.9736
2	**1.0000**	1.0000	1.0000	1.0000	1.0000	1.0000	1.0000	1.0000	1.0000	1.0000	0.3924

**Fig 8:**
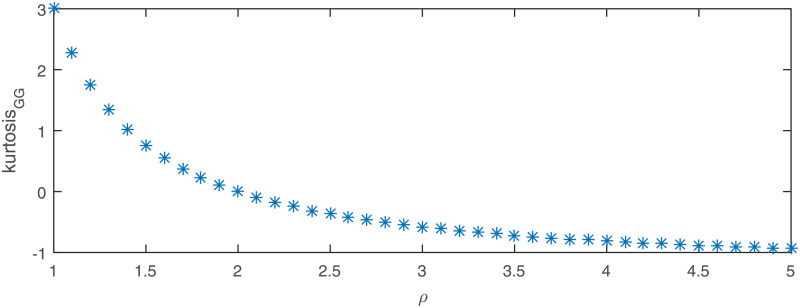
Theoretical excess kurtosis for the generalized Gaussian distribution with respect to the *ρ* parameter.

The second considered distribution is the mixed Gaussian which is also a member of the platykurtic class. Here we assume *μ*_1_ = 1, *σ*_1_ = 1 and *σ*_2_ = 1 and analyze the power of the test by changing the *μ*_2_ parameter. As it is explained in the Appendix, there is one to one correspondence between the *μ*_2_ parameter and the excess kurtosis of the mixed Gaussian distribution (see formula (16)). Therefore, in Fig 8 we present the power of the test with respect to the excess kurtosis and compare it with the power of the common tests for normality. As previously, we can see that the proposed test is clearly superior to other tests for *N* > 100 while its performance diminishes for sample size *N* = 50. Again, we can see that the least performing tests in this study are the KS test, the *χ*^2^ test, and the JB test, especially for short samples.

The next two considered distributions, namely *α*-stable and Student’s *t*-distributions belong to the class of leptokurtic distributions. We note that the *α*-stable distribution is difficult to differentiate from the normal distribution when *α* is close to two and the same is true for the Student’s *t* distribution when number of degrees of freedom is large [31, 32, 17].

**Fig 9:**
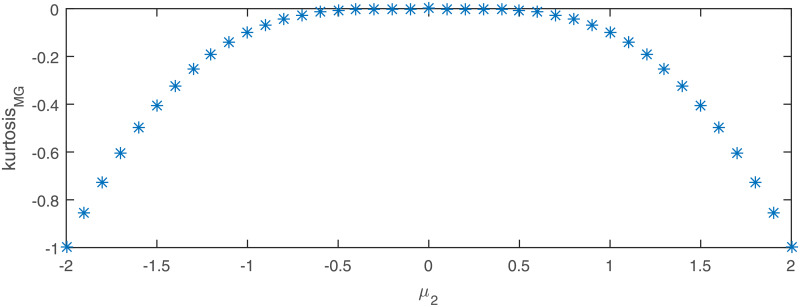
Theoretical excess kurtosis for the mixed Gaussian distribution with *m* = 2, *p*_1_ = *p*_2_ = 0.5, *μ*_1_ = 0, $\sigma_{1}^{2}=\sigma_{2}^{2}=1$ as a function of the parameter *μ*_2_.

In Fig 9 we consider the symmetric *α*-stable distribution with *σ* = 1. Since for the *α*-stable distribution excess kurtosis is infinite, in this study we analyze the power of the test with respect to the stability index *α* and compare the test performance with results of the classical tests. We can see that the situation for the leptokurtic distributions is different than for the platykurtic. We can clearly identify two groups of the tests. The first group, which consists of the introduced test, JB, SW and DP tests performs much better than the second group with the rest of the tests (only for *N* = 50 the introduced test visibly falls behind the top three tests but is still superior to others). It seems that the SW test has the best overall performance, and the proposed test is the last in the first group for smaller and moderate samples but for *N* = 1000 it behaves very similarly to the SW.

**Fig 10:**
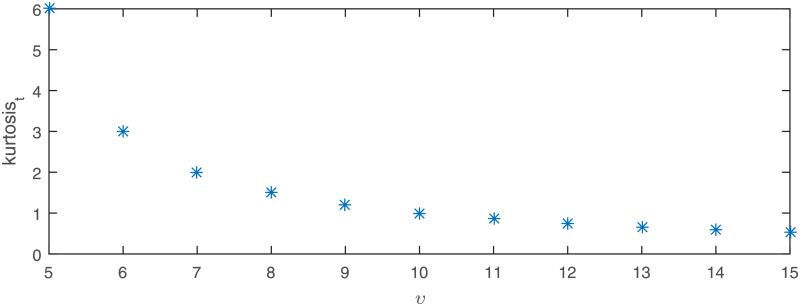
Theoretical excess kurtosis for Student’s *t*-distribution as a function of the parameter *ν*.

The last considered distribution is the Student’s *t*. In Fig 10 the power of the proposed test for normality is presented as a function of the number of the degrees of freedom since for the Student’s *t*-distribution the excess kurtosis is finite only if number of degrees of freedom exceeds 4. As in the previous cases, the power results are compared to those of the common tests for normality. We can see that the situation is very similar to the observed for the *α*-stable distribution. We can observe two groups of tests. The introduced test belongs to a selected group of test that perform much better than the rest of the distributions (again only for *N* = 50 it visibly falls behind the leaders). In this case, it seems that the JB test is superior for most of the cases. The proposed test is the last in the first group for smaller and moderate samples but for *N* = 1000 it becomes even better than the JB test.

In the Appendix in Tables 2–21 we present powers of the considered normality tests with the highlighted best results also for *N* = 20. The situation for *N* = 20 is similar to the case *N* = 50 with two striking differences: the *χ*^2^ test fails to reject simulated samples from the generalized Gaussian, mixed Gaussian and *α*-stable distributions, and the CF test significantly improves its performance being the clear winner for the generalized Gaussian and mixed Gaussian distributions, see also Figs 3–6.

In order to analyze the influence of the *n* parameter on the effectiveness of the proposed test, in the Appendix we present the comparison of the power of the test for *n* = 2 and *n* = 3 for all considered distributions and sample sizes. In Tables 22–25 we demonstrate the power of the test and in each considered case we highlight the best results. We observe that for the platykurtic distributions generally the test for *n* = 2 outperforms the test for *n* = 3, especially for larger sample sizes and excess kurtosis much smaller than zero. For the leptokurtic distributions in both cases, namely for *n* = 2 and *n* = 3, the power of the test is comparable.

**Table 22:** Comparison of the power of the test for *n* = 2 and *n* = 3—GG distribution.

	*N* = 20		*N* = 50		*N* = 100		*N* = 200		*N* = 1000	
kurtosis	*n* = 2	*n* = 3	*n* = 2	*n* = 3	*n* = 2	*n* = 3	*n* = 2	*n* = 3	*n* = 2	*n* = 3
0.0000	0.0446	**0.0516**	0.0420	**0.0458**	0.0452	**0.0464**	**0.0442**	0.0428	0.0458	**0.0478**
-0.0934	**0.0458**	0.0434	0.0406	**0.0448**	**0.0544**	0.0462	**0.0604**	0.0598	**0.0882**	0.0882
-0.1753	**0.0468**	0.0466	0.0476	**0.0484**	**0.0588**	0.0502	0.0754	**0.0778**	0.2268	**0.2278**
-0.2475	0.0454	**0.0474**	0.0438	**0.0460**	**0.0712**	0.0496	**0.1204**	0.1160	**0.424**	0.4220
-0.3116	0.0438	**0.0462**	0.0460	**0.0480**	**0.081**	0.0630	**0.1576**	0.1490	**0.6388**	0.6384
-0.3688	0.0434	**0.0466**	**0.0514**	0.0470	**0.108**	0.0682	**0.2156**	0.2084	**0.8042**	0.8024
-0.4202	0.0472	**0.0476**	**0.0534**	0.0448	**0.1298**	0.0848	**0.2738**	0.2666	**0.9176**	0.9168
-0.4666	**0.0484**	0.0482	**0.0592**	0.0526	**0.1758**	0.1028	**0.3514**	0.3374	0.9674	**0.9682**
-0.5086	**0.0446**	0.0446	**0.0604**	0.0578	**0.1844**	0.1064	**0.4218**	0.4106	**0.9882**	0.9882
-0.5468	**0.0468**	0.0434	**0.0654**	0.0614	**0.2188**	0.1310	**0.491**	0.4636	**0.9992**	0.9992
-0.5816	0.0514	**0.0548**	**0.0636**	0.0632	**0.256**	0.1330	**0.5584**	0.5386	**0.9996**	0.9996
-0.6135	**0.0454**	0.0548	**0.0648**	0.0614	**0.2772**	0.1582	**0.6296**	0.6070	**1.0000**	1.0000
-0.6428	**0.0468**	0.0468	**0.0768**	0.0680	**0.3118**	0.1838	**0.6878**	0.6546	**1.0000**	1.0000
-0.6698	**0.0468**	0.0452	**0.0810**	0.0726	**0.3496**	0.1976	**0.7318**	0.7020	**1.0000**	1.0000
-0.6948	0.0482	**0.0512**	**0.0816**	0.0708	**0.3804**	0.2182	**0.7672**	0.7460	**1.0000**	1.0000
-0.7179	0.0490	**0.0524**	**0.0888**	0.0762	**0.4096**	0.2276	**0.8202**	0.8018	**1.0000**	1.0000
-0.7394	**0.0522**	0.0502	**0.0846**	0.0722	**0.4474**	0.2526	**0.844**	0.8212	**1.0000**	1.0000
-0.7593	**0.0528**	0.0518	**0.0912**	0.0816	**0.4714**	0.2726	**0.8736**	0.8616	**1.0000**	1.0000
-0.7779	**0.0498**	0.0490	**0.0966**	0.0766	**0.5074**	0.2824	**0.8916**	0.8764	**1.0000**	1.0000
-0.7953	**0.0592**	0.0494	**0.0968**	0.0840	**0.5238**	0.3122	**0.9118**	0.9018	**1.0000**	1.0000
-0.8116	**0.0562**	0.0532	**0.0994**	0.0866	**0.5494**	0.3244	**0.9296**	0.9200	**1.0000**	1.0000
-0.8268	**0.0542**	0.0520	**0.1024**	0.0804	**0.5748**	0.3436	**0.9398**	0.9262	**1.0000**	1.0000
-0.8411	**0.0544**	0.0444	**0.1046**	0.0818	**0.5922**	0.3704	**0.9446**	0.9330	**1.0000**	1.0000
-0.8546	**0.0564**	0.0494	**0.1100**	0.0810	**0.6122**	0.3668	**0.9498**	0.9420	**1.0000**	1.0000
-0.8672	**0.0560**	0.0512	**0.1146**	0.0894	**0.6228**	0.3912	**0.9584**	0.9512	**1.0000**	1.0000
-0.8792	**0.0612**	0.0544	**0.1126**	0.0842	**0.6606**	0.3954	**0.9694**	0.9624	**1.0000**	1.0000

**Table 23:** Comparison of the power of the test for *n* = 2 and *n* = 3—MG distribution.

	*N* = 20		*N* = 50		*N* = 100		*N* = 200		*N* = 1000	
kurtosis	*n* = 2	*n* = 3	*n* = 2	*n* = 3	*n* = 2	*n* = 3	*n* = 2	*n* = 3	*n* = 2	*n* = 3
-0.0380	0.0466	**0.0470**	0.0442	**0.0482**	**0.0460**	0.0378	0.0464	**0.0450**	**0.0540**	0.0538
-0.0567	0.0462	**0.0482**	0.0480	**0.0508**	**0.0436**	0.0396	**0.0490**	0.0450	0.0650	**0.0652**
-0.0800	0.0456	**0.0520**	0.0398	**0.0432**	**0.0536**	0.0474	0.0492	**0.0506**	**0.0774**	0.0772
-0.1079	0.0436	**0.0474**	0.0394	**0.0412**	0.0408	**0.0436**	0.0546	**0.0576**	0.1010	**0.1016**
-0.1401	0.0428	**0.0506**	0.0448	**0.0490**	**0.0544**	0.0470	0.0638	**0.0674**	0.1406	**0.1530**
-0.1764	0.0412	**0.0458**	0.0418	**0.0454**	**0.0586**	0.0430	0.0730	**0.0736**	**0.2216**	0.2208
-0.2163	0.0380	**0.0466**	0.0414	**0.0450**	**0.0626**	0.0476	**0.0996**	0.0932	0.3260	**0.3262**
-0.2592	**0.0438**	0.0412	0.0400	**0.0434**	**0.0720**	0.0540	**0.1232**	0.1174	0.4624	**0.4642**
-0.3046	**0.0456**	0.0454	0.0398	**0.0482**	**0.0860**	0.0622	**0.1592**	0.1506	**0.6178**	0.6158
-0.3519	0.0462	**0.0476**	**0.0506**	0.0490	**0.1056**	0.0694	**0.2042**	0.1932	**0.7558**	0.7548
-0.4005	0.0460	**0.0494**	0.0546	**0.0550**	**0.1300**	0.0850	**0.2576**	0.2480	**0.8812**	0.8808
-0.4501	**0.0482**	0.0444	**0.0556**	0.0542	**0.1622**	0.0980	**0.3454**	0.3318	**0.9498**	0.9492
-0.5000	0.0444	**0.0468**	**0.0612**	0.0590	**0.1940**	0.1144	**0.4164**	0.4012	**0.9824**	0.9818
-0.5499	**0.0478**	0.0462	**0.0674**	0.0570	**0.2394**	0.1440	**0.5082**	0.4884	**0.9952**	0.9952
-0.5995	0.0512	**0.0528**	**0.0704**	0.0690	**0.2840**	0.1664	**0.5828**	0.5706	**0.9988**	0.9986
-0.6485	**0.0596**	0.0594	**0.0890**	0.0778	**0.3468**	0.2122	**0.6896**	0.6736	**1.0000**	1.0000
-0.6966	0.0546	**0.0558**	**0.0894**	0.0830	**0.3948**	0.2412	**0.7692**	0.7512	**1.0000**	1.0000
-0.7436	**0.0656**	0.0566	**0.1154**	0.0932	**0.4790**	0.2988	**0.8338**	0.8184	**1.0000**	1.0000
-0.7894	**0.0728**	0.0569	**0.1234**	0.1030	**0.5284**	0.3404	**0.8800**	0.8672	**1.0000**	1.0000
-0.8339	**0.0712**	0.0640	**0.1434**	0.1054	**0.5852**	0.3980	**0.9150**	0.9064	**1.0000**	1.0000
-0.8769	**0.0858**	0.0698	**0.1520**	0.1254	**0.6532**	0.4572	**0.9444**	0.9346	**1.0000**	1.0000
-0.9185	**0.0910**	0.0788	**0.1876**	0.1458	**0.7030**	0.5100	**0.9632**	0.9584	**1.0000**	1.0000
-0.9586	**0.0924**	0.0770	**0.1912**	0.1478	**0.7542**	0.5684	**0.9734**	0.9698	**1.0000**	1.0000

**Table 24:** Comparison of the power of the test for *n* = 2 and *n* = 3 − *α*-stable distribution.

	*N* = 20		*N* = 50		*N* = 100		*N* = 200		*N* = 1000	
*α*	*n* = 2	*n* = 3	*n* = 2	*n* = 3	*n* = 2	*n* = 3	*n* = 2	*n* = 3	*n* = 2	*n* = 3
2	0.0462	**0.0530**	0.0446	**0.0512**	**0.0460**	0.0456	**0.0532**	0.0518	0.0504	**0.0518**
1.99	0.0518	**0.0570**	**0.0678**	0.0674	**0.0876**	0.0846	**0.1198**	0.1182	0.3014	**0.3042**
1.98	0.0608	**0.0634**	0.0854	**0.0866**	**0.1276**	0.1224	**0.1836**	0.1814	**0.5310**	0.5310
1.97	**0.0822**	0.0784	**0.1128**	0.1118	**0.1752**	0.1654	**0.2604**	0.2562	0.6926	**0.6944**
1.96	**0.0756**	0.0718	0.1340	**0.1354**	**0.2026**	0.1990	**0.3136**	0.3132	0.7862	**0.7890**

**Table 25:** Comparison of the power of the test for *n* = 2 and *n* = 3—Student’s t distribution.

	*N* = 20		*N* = 50		*N* = 100		*N* = 200		*N* = 1000	
*ν*	*n* = 2	*n* = 3	*n* = 2	*n* = 3	*n* = 2	*n* = 3	*n* = 2	*n* = 3	*n* = 2	*n* = 3
36	0.0582	**0.0660**	0.0672	**0.0694**	**0.0780**	0.0730	0.0908	**0.0928**	0.2360	**0.2392**
34	0.0520	**0.0626**	0.0556	**0.0606**	**0.0748**	0.0710	**0.0840**	0.0838	0.2380	**0.2414**
30	0.0526	**0.0680**	0.0650	**0.0730**	**0.0922**	0.0900	0.1020	**0.1012**	0.2890	**0.2940**
26	0.0578	**0.0776**	0.0718	**0.0782**	**0.0954**	0.0900	0.1142	**0.1146**	0.3558	**0.3622**
22	0.0614	**0.0844**	0.0770	**0.0826**	**0.1096**	0.1022	**0.1382**	0.1382	0.4568	**0.4590**
20	0.0630	**0.0796**	**0.0832**	0.0776	**0.1096**	0.1004	**0.1504**	0.1474	0.5086	**0.5116**
18	0.0582	**0.0790**	0.0874	**0.0900**	**0.1226**	0.1184	**0.1716**	0.1656	0.5972	**0.6050**
16	0.0660	**0.0872**	0.0920	**0.0928**	**0.1388**	0.1324	**0.2036**	0.2024	0.6660	**0.6696**
14	0.0698	**0.0968**	**0.1098**	0.1068	**0.1646**	0.1568	**0.2520**	0.2466	0.7772	**0.7808**
12	0.0764	**0.1142**	**0.1270**	0.1268	**0.1978**	0.1904	**0.3050**	0.2982	0.8710	**0.8724**
10	0.0946	**0.1348**	**0.1568**	0.1494	**0.2626**	0.2516	**0.4088**	0.4000	0.9462	**0.9474**
8	0.1044	**0.1496**	**0.2016**	0.1922	**0.3376**	0.3240	**0.5438**	0.5334	**0.9934**	0.9932
6	0.1430	**0.1970**	**0.2718**	0.2554	**0.4680**	0.4506	**0.7216**	0.7148	**0.9998**	0.9998
4	0.2070	**0.2928**	**0.4574**	0.4388	**0.7236**	0.7078	**0.9340**	0.9302	**1.0000**	1.0000
2	0.4696	**0.5728**	**0.8378**	0.8168	**0.9834**	0.9820	**0.9996**	0.9994	**1.0000**	1.0000

## Application to real time series

In order to demonstrate how the proposed methodology can be applied to real data, in this section we consider two illustrative datasets from a collection of over 1300 datasets that were originally distributed alongside the R environment [33]. The inclusion criteria for a dataset to be considered an illustrative example in our study were: sufficient sample size, lack of obvious trend, lack of obvious correlation and platykurtosis. When necessary, data differentiation was considered to arrive at weak stationarity. The first dataset (Data 1) is related to oil investments. In the collection the data are under the name “Oil Investment”. For the analysis, we took the variable “waterd”, which describes the depth of the sea in metres and we examine the first 50 available observations. A detailed description of the data can be found in Ref. [34]. The second dataset (Data 2) corresponds to the non-experimental “control” group, used in various studies of the effect of a labor training program. The time series is titled “Labour Training Evaluation Data”. To the analysis we took the first 200 observations of the differentiated time series “re78“, which describes the real earnings in the year of 1978 [35, 36, 37, 38].

**Fig 11:**
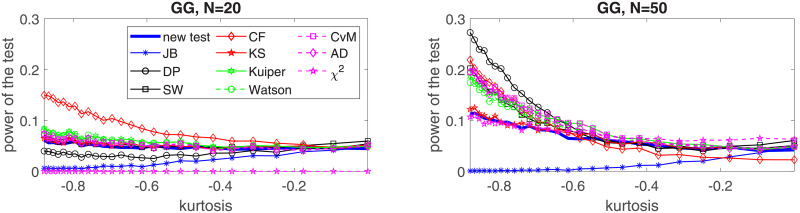
Comparison of the power of the introduced test and standard tests for normality for *N* = 20 and *N* = 50 for the generalized Gaussian distribution with respect to the excess kurtosis.

The two considered datasets are presented in Fig 11. For both considered time series we apply the proposed test to ascertain whether the hypothesis of normality can be rejected. The testing procedure is illustrated in Fig 2 Schema 1. First, for a dataset of size *N* we calculate the value of the test statistic $H_{2}^{max}$, see formula (10). Then, we check if the calculated value $H_{2}^{max}$ falls between lower and upper critical values constructed for the samples of size *N* at a given significance level, see Table 1. We reject the hypothesis of normal distribution if the value of the test statistic is smaller than the lower critical value *Q*_1_ or higher than the upper critical value *Q*_2_. The results of testing the hypothesis of normality for the two considered real datasets with the proposed test as well as the other considered here normality tests are presented in Table 26. We present the following information about the results of the tests at the significance levels *c* = 0.01 and *c* = 0.05: 0 means the hypothesis of normality was not rejected whereas 1 implies the hypothesis of normality was rejected. For the new test introduced in this paper we also depict the test statistic values in the parentheses.

**Table 26:** Results of the new test and other considered here tests for normality for two real-world datasets at significance levels of *c* = 0.01 and *c* = 0.05. Tests taken into consideration: the new test proposed in this paper, Jarque-Bera (JB) test [5], D’Agostino-Pearson (DP) test [4], Shapiro-Wilk (SW) test [3], test based on the empirical characteristic function (CF) [22], Kolmogorov-Smirnov (KS) test [2], Kuiper test [6], Watson test [7], Cramer-von Mises (CvM) test [8], Anderson-Darling (AD) test [9] and *χ*^2^ goodness-of-fit test [1]. “0” means that the normality is not rejected, “1”—it is rejected. In parentheses values of the test statistic for the new test are presented.

*dataset*	*N*	*kurtosis*	*c*	*new test*	*JB*	*DP*	*SW*	*CF*	*KS*	*Kuiper*	*Watson*	*CvM*	*AD*	*χ*^2^
Data 1	50	−0.9351	0.01	1 (0.1022)	0	0	0	0	0	0	0	0	0	1
			0.05	1 (0.1022)	0	0	1	0	1	1	1	1	1	1
Data 2	200	−0.6096	0.01	0 (0.1242)	0	0	0	0	0	0	0	0	0	0
			0.05	1 (0.1242)	0	0	0	0	0	0	0	0	0	0

For Data 1 we can observe that the new test rejects the hypothesis of normality at both significance levels: 1% and 5%. At significance level 5% the same result is obtained for SW, KS, Kuiper, Watson, CvM, AD and *χ*^2^ tests. The JB, DP and CF tests do not reject the hypothesis of normality although the empirical kurtosis is smaller than zero (see Table 26). At significance level of 1% the introduced and *χ*^2^ tests are the only ones that lead to the rejection of the null hypothesis. Interestingly, for Data 2, only the new test rejects the hypothesis of normality at the significance level of 5%.

## Conclusions

Developing an omnibus test for normality of a random sample is a challenging and important task in signal processing that is particularly difficult for symmetric alternatives and those that are close to the normal distribution. We examined the behavior of the Edgeworth expansion when the distribution of a random sample deviates from the normal assumption. Then, by appropriately utilizing the second term of this expansion we designed a novel test on normality that can be treated as omnibus.

The test’s performance, evaluated via Monte Carlo simulations, showed superior performance to those exhibited by other statistical tests for normality, particularly for the case of platykurtic distributions and sample sizes greater or equal to 100. For these distributions, the proposed test in almost all cases had the highest power.

For the leptokurtic distributions the situation was different, but still the proposed test was among the best. For this class of distributions, we were able to identify two groups of the tests. The power of the proposed test was shown to belong to the group of powerful tests that include the well-known D’Agostino-Pearson, Shapiro-Wilk and Jarque-Bera tests. For the largest size of the sample it even surpassed these top competitors.

We also showed the efficacy of the introduced test by studying two datasets from the open R language data repository. We compared the results of the proposed test and those of the other considered here normality tests. It is evident that for the first dataset the new test was among a few which led to the rejection of the hypothesis of normality at significance level of 5%. At the level of 1%, only the proposed and the *χ*^2^ tests rejected normality. For the second dataset the new test was the only one which rejected the hypothesis of normality.

Finally, we also note that the test is relatively easy to use. The introduced statistic is computationally simple. To conduct the test one needs to calculate critical values for a given significance level. In the paper we presented a simple algorithm to calculate these values and provided the critical values for typical sample sizes and significance levels.

## Appendix

We review some of the classical distributions used in this work, which belong to the platykurtic and leptokurtic families of distributions.

### Platykurtic distributions

#### 1. Generalized Gaussian distribution

The generalized Gaussian GG(*μ*, *β*, *ρ*) is characterized by the probability density function given by the formula [39, 40]

$$\begin{array}{c} f\left(x\right)=\frac{\beta^{1/2}}{2\Gamma (1+1/\rho )}e^{-\beta^{\rho /2}{\left|x-\mu \right|}^{\rho }},\;x\in R, \end{array}$$ (13)

where *μ* ∈ *R*, *β*, *ρ* > 0. The *ρ* parameter controls how heavy is the tail. The excess kurtosis in this case takes the form

$$\begin{array}{c} kurtosis_{GG}=\frac{\Gamma (5/\rho )\Gamma (1/\rho )}{\Gamma {\left(3/\rho \right)}^{2}}-3. \end{array}$$ (14)

**Fig 12:**
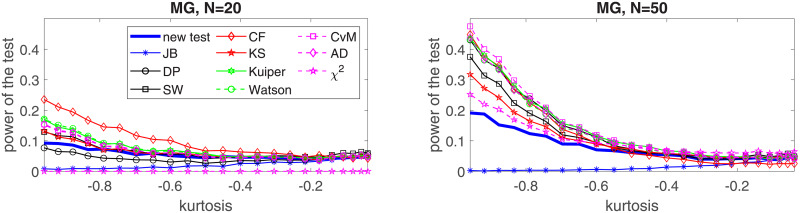
Comparison of the power of the introduced test and standard tests for normality for *N* = 20 and *N* = 50 for the mixed Gaussian distribution with respect to the excess kurtosis.

In Fig 12 we present the theoretical excess kurtosis of GG distribution along the *ρ* parameter. The GG distribution can either be leptokurtic or platykurtic, depending on the parameter *ρ*. Nevertheless, in this paper we focus on the platykurtic region of the parameter *ρ*.

#### 2. Mixture of Gaussian distributions

A random variable *M* follows a mixture of *m* Gaussian distributions if its PDF has the form [41, 42]

$$\begin{array}{c} f\left(x\right)=\sum_{i=1}^{m}p_{i}f_{i}\left(x\right),\;x\in R, \end{array}$$ (15)

where *p*_*i*_ ≥ 0, $\sum_{i=1}^{m}p_{i}=1$ and *f*_*i*_(⋅) is a PDF of a normally distributed random variable with mean *μ*_*i*_ and variance $\sigma_{i}^{2}$.

In this paper we take under consideration the simplest case, namely *m* = 2, *p*_1_ = *p*_2_ = 0.5, *μ*_1_ = 0, $\sigma_{1}^{2}=\sigma_{2}^{2}=1$ and consider the MG distribution only in terms of the *μ*_2_ parameter. In this case the excess kurtosis is given by

$$\begin{array}{c} kurtosis_{MG}=\frac{-\mu_{2}^{4}}{8{\left(1+\frac{1}{4}\mu_{2}^{2}\right)}^{2}}. \end{array}$$ (16)

The above formula can be proved as follows. Using the PDF of MG distribution given in (15) one can show that

$$\begin{array}{c} E\left(M\right)=\frac{1}{2}\mu_{2}^{2},\;E\left(M^{2}\right)=1+\frac{1}{2}\mu_{2}^{2}. \end{array}$$

Thus

$$\begin{array}{c} Var\left(M\right)=1+\frac{1}{4}\mu_{2}^{2}. \end{array}$$

Further

$$\begin{array}{c} E{\left(M-E\left(M\right)\right)}^{4}=E\left(M^{4}\right)-4E\left(M^{3}\right)E\left(M\right)+6E\left(M^{2}\right){\left(E\left(M\right)\right)}^{2}-3{\left(E\left(M\right)\right)}^{4}. \end{array}$$

Using the formula for PDF of the random variable *M* we obtain

$$\begin{array}{c} E\left(M^{4}\right)=3+3\mu_{2}^{2}+\frac{1}{2}\mu_{2}^{4},\;E\left(M^{3}\right)=\frac{1}{2}\mu_{2}^{3}+\frac{3}{2}\mu_{2}. \end{array}$$

Thus, finally the excess kurtosis for the MG random variable when *m* = 2, $p_{1}=p_{2}=\frac{1}{2}$, *m*_1_ = 0 and *σ*_1_ = *σ*_2_ = 1 takes the form

$$\begin{array}{c} kurtosis_{MG}=\frac{E({\left(M-EM\right)}^{4})}{Var(M)}-3=\frac{3+\frac{3}{2}\mu_{2}^{2}+\frac{1}{16}\mu_{2}^{4}}{{\left(1+\frac{1}{4}\mu_{2}^{2}\right)}^{2}}-3=\frac{-\mu_{2}^{4}}{8{\left(1+\frac{1}{4}\mu_{2}^{2}\right)}^{2}}. \end{array}$$

**Fig 13:**
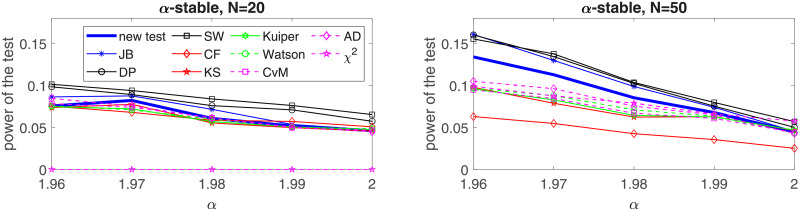
Comparison of the power of the introduced test and standard tests for normality for *N* = 20 and *N* = 50 for the *α*-stable distribution with respect to the *α* parameter.

As one can see in the considered case the MG distribution is platykurtic. In Fig 13 we present the excess kurtosis of MG distribution with *m* = 2, *p*_1_ = *p*_2_ = 0.5, *μ*_1_ = 0, $\sigma_{1}^{2}=\sigma_{2}^{2}=1$ with respect to the *μ*_2_ parameter.

### Leptokurtic distributions

#### 1. Student’s *t*-distribution

The Student’s *t*-distribution is defined through its PDF given by the formula [43, 44]

$$\begin{array}{c} f\left(x\right)=\frac{\Gamma (\frac{\nu +1}{2})}{\Gamma (\frac{\nu }{2})\sqrt{\nu \pi }}{\left(1+\frac{x^{2}}{\nu }\right)}^{-\frac{\nu +1}{2}},\;x\in R \end{array}$$ (17)

where the parameter *ν* > 0 is called the number of degrees of freedom. In the above definition Γ(⋅) is the gamma function. The excess kurtosis for Student’s *t*-distribution is defined only for *ν* > 4 and takes the form

$$\begin{array}{c} kurtosis_{t}=\frac{6}{\nu -4}. \end{array}$$ (18)

**Fig 14:**
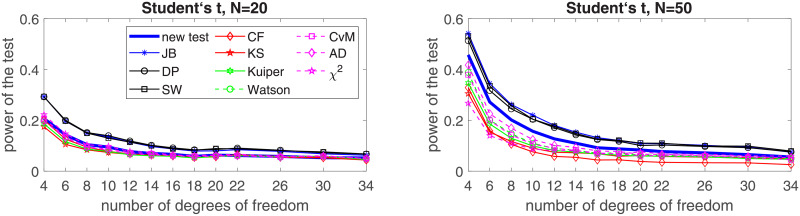
Comparison of the power of the introduced test and standard tests for normality for *N* = 20 and *N* = 50 for the Student’s *t*-distribution with respect to the number of degrees of freedom.

In Fig 14 we show the theoretical excess kurtosis for Student’s *t*-distribution with respect to the *ν* parameter.

#### 2. *α*-stable distribution

The *α*-stable random variable with parameters *α*, *σ*, *β* and *μ* is defined through its characteristic function in the following way [45]

$$\begin{array}{ccc} \Phi (k) & = & \left\{ \begin{array}{ccc} \text{exp}\{-\sigma^{\alpha }|k{|}^{\alpha }\left(1-i\beta \text{sgn}\left(k\right)\text{tan}\frac{\pi \alpha }{2}\right)+ik\mu \} & \text{if} & \alpha \neq 1,\\ \text{exp}\{-\sigma |k|(1-i\beta \frac{2}{\pi }\text{sgn}\left(k\right)\text{ln}|k|)+ik\mu )\} & \text{if} & \alpha =1, \end{array}\right. \end{array}$$ (19)

where 0 < *α* ≤ 2 is the stability index, *σ* > 0 is the scale parameter, −1 ≤ *β* ≤ 1 is the skewness parameter and *μ* ∈ *R* is the location parameter. The explicit formula for the PDF of *α*-stable random variable is not given in an elementary form, except for the three cases: normal, Cauchy and Lévy distributions. In this work, distributions with the stability index *α* close to two (i.e., the case of normal distribution) are studied in detail.

#### Comparison of the power of the test for *n* = 2 and *n* = 3

In this part we present a comparison of the power of the test for two cases: *n* = 2 and *n* = 3. In Tables 22–25 we highlight the best results for the considered cases.

#### Comparison of the computational times

In this part we present a comparison of the computational time of the proposed normality test with the computational times of the other considered tests. Here we only present the running times for one exemplary distribution, namely the Gaussian, and one sample size, namely *N* = 1000. The power of each test was calculated on the basis of 5000 simulations. In Table 27 we depict mean computational times calculated as the time needed to evaluate the power of the test divided by the number of the Monte Carlo simulations (in our case 5000). We note that the tests based on the empirical distribution function (EDF tests) seem to be unusually slow. This is due to the fact we calculated powers by evaluating *p*-values for all samples by means of Monte Carlo simulations as advocated by Ross [46] for goodness of fit testing for the case of unspecified parameters.

**Table 27:** Comparison of the mean computational time (in seconds) for the considered tests for the sample from Gaussian distribution of size *N* = 1000. The powers of the tests are are calculated based on the 5000 Monte Carlo simulations.

*New* *test*	*JB*	*DP*	*SW*	*CF*	*TestsbasedonEDF*	*χ*^2^
0.0022	0.0011	0.0002	0.0006	0.0152	∼0.1	0.0004

#### Critical values of test

In this part we present critical values of the introduced test for the considered five sample sizes (*N* = 20, 50, 100, 200, 1000) and two significance levels *c*: 1% and 5%, see Table 1.

#### Comparison of the powers of the tests for normality

In this part we present a comparison of the powers of the normality tests considered in this paper for four distributions and for five sample sizes (*N* = 20, 50, 100, 200, 1000). The results are presented in Tables 2–21, where we have highlighted the best results.
